# Characterising the Periodontal Granulation Tissue Using scRNAseq

**DOI:** 10.1111/jcpe.70048

**Published:** 2025-10-07

**Authors:** Wentao Zhu, Kathy Fung, Pawan Dhami, Paul Sharpe, Jan Krivanek, Luigi Nibali, Cheng Zhang, Vitor C. M. Neves

**Affiliations:** ^1^ Department of Women's and Children's Health Karolinska Institutet Stockholm Sweden; ^2^ Science for Life Laboratory KTH – Royal Institute of Technology Stockholm Sweden; ^3^ NIHR BRC Genomics Research Platform, Guy's and St Thomas' NHS Foundation Trust, King's College London School of Medicine London UK; ^4^ Centre for Craniofacial and Regenerative Biology, FoDOCS, King's College London London UK; ^5^ Institute of Animal Physiology and Genetics Brno Czech Republic; ^6^ Department of Histology and Embryology, Faculty of Medicine Masaryk University Brno Czech Republic; ^7^ Periodontology Unit, Centre for Host‐Microbiome Interactions, FoDOCS, King's College London London UK; ^8^ Faculty of Life Sciences & Medicine, School of Immunology & Microbial Sciences, James Black Centre, King's College London The Roger Williams Institute of Liver Studies London UK; ^9^ The School of Clinical Dentistry University of Sheffield Sheffield UK; ^10^ Healthy Lifespan Institute University of Sheffield Sheffield UK

## Abstract

**Aim:**

To investigate the cellular composition and molecular mechanisms of periodontal granulation tissue formation using single‐cell RNA sequencing (scRNA‐seq), aiming to enhance the understanding of periodontal disease pathogenesis and identify potential targets for regenerative therapies.

**Materials and Methods:**

Granulation tissue samples were collected from patients undergoing periodontal surgery (*n* = 3). Fresh tissues were processed into single‐cell suspensions and subjected to scRNA‐seq. The data were integrated and compared with existing datasets from healthy gingiva and periodontal ligament. Computational analyses were performed and validated through immunofluorescence staining.

**Results:**

Ten distinct cell clusters were identified across the samples. Granulation tissue exhibited a higher abundance of immune cells compared to healthy tissues. A novel endothelial cell subpopulation, exclusive to granulation tissue, was discovered and characterised by *NOTCH3* expression and involvement in ossification pathways. Additionally, granulation tissue fibroblast subpopulations demonstrated a progenitor‐like state, characterised by extracellular matrix reorganisation and low differentiation, similar to cancer‐associated fibroblasts.

**Conclusion:**

This study identified a novel endothelial subpopulation offering new insights into the disease's pathogenesis and presenting potential targets for regenerative therapies. These findings will help advance the understanding of periodontal disease granulation tissue formation and provide information for the development of materials to modulate specific cellular pathways to improve periodontal disease management.

## 1 Introduction

The gingiva, characterised by a keratinized stratified squamous epithelium and an underlying connective tissue matrix, is an actively proliferating tissue, serving as the frontline defence against oral diseases. This tissue is responsible for maintaining biological equilibrium amidst various microenvironmental challenges; however, inadequate oral hygiene practices, facilitating the accumulation and calcification of plaque—a bacterial biofilm—can disrupt this delicate balance (Kinane et al. 2017). Consequently, host immune responses precipitate dysbiosis within the oral microbiota, leading to periodontal disease, comprising a spectrum of inflammatory conditions afflicting the periodontal tissues and culminating in irreversible damage to the supporting structures of the teeth (Van Dyke et al. 2020).

The sequelae of periodontal disease manifest as the destruction of attachment, including bone and ligaments, accompanied by a transformation of gingival tissue from its anatomically defined state to fibrous and inflamed granulation tissue (Apatzidou et al. 2018). Traditionally, granulation tissue has been excised during periodontal therapy (Kwon et al. 2021). However, emerging evidence suggests that this tissue may harbour cells with regenerative and ossification potential, thus prompting reconsideration of its therapeutic implications (Gousopoulou et al. 2023).

Recent clinical research suggests that retaining the granulation results in similar bone infill to surgeries that remove them and add biomaterials (Apatzidou et al. 2021; Adam et al. 2022). Therefore, this raises the question of whether there is a need to remove granulation tissue. Recent data have demonstrated the transcriptomic profile of granulation tissue (Sam et al. 2025); however, the cellular composition and contributions from the periodontal tissues to the formation of granulation tissue remain poorly understood. This knowledge gap underscores the challenges associated with regenerating the periodontium in patients with periodontitis, making clinical choices both challenging and unpredictable. Therefore, characterisation of the granulation tissue cell components and their origins can have profound implications for understanding the pathogenesis of periodontal disease and may provide new avenues for regenerative therapies.

Over a decade ago, a new tool to understand transcriptome dynamics was developed. Single‐cell RNA‐sequencing (scRNAseq) technologies can quantify intra‐population heterogeneity and enable the study of cell states and transitions at very high resolution, potentially revealing cell subtypes or gene expression dynamics that are masked in bulk, population‐averaged measurements. To date, this tool has generated extensive knowledge about the pathogenesis of periodontitis, including the gingival molecular changes from health to disease (Caetano et al. 2021), the osteoimmunology of the disease (Chen et al. 2022), the immune compartment reaction of the periodontium (Liu et al. 2023), the role of macrophages in the disease in health and type 2 diabetes (Agrafioti et al. 2022) and the characterisation of the dental pulp and periodontal ligament cell composition (Yang et al. 2024; Pagella, De Vargas Roditi, et al. 2021). However, the cellular composition and the origin of granulation tissue that is removed during surgery remain unclear.

Therefore, this research aims to use scRNAseq to identify the composition of granulation tissue, uncovering its cellular underpinnings, and to clarify how healthy gingiva and the periodontal ligament (PDL) transition into granulation tissue. Ultimately, the objective is to find novel cellular and molecular targets for regenerative and reparative periodontal therapy.

## 2 Materials and Methods

**FIGURE 1:**
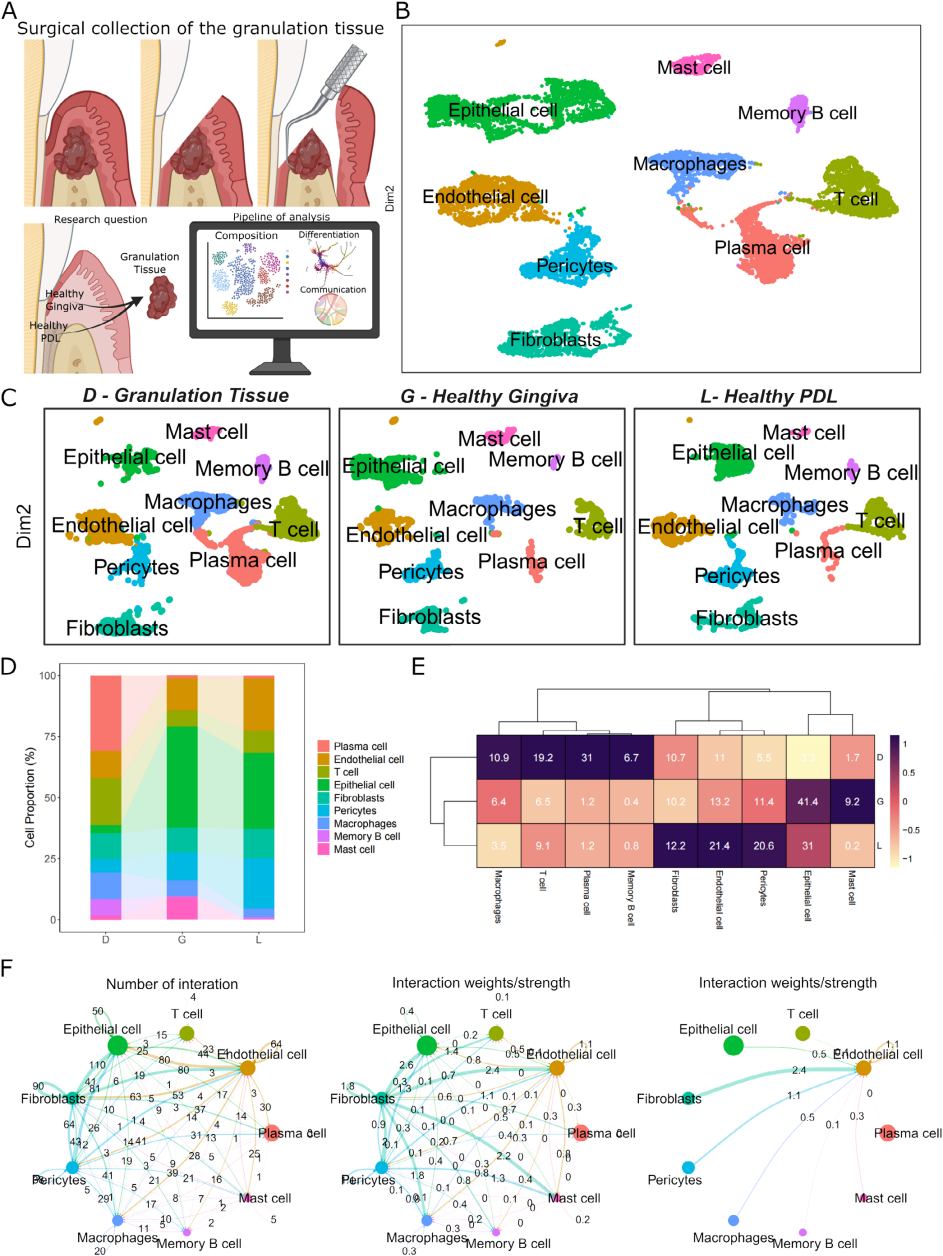
Characterising the populations of periodontal tissues. (A) Schematic overview of the tissue harvesting technique of this study, research question ‘What is the composition and origin of granulation tissues?’ and pipeline of analysis. (B) Uniform manifold approximation and projection (UMAP) representation showing nine cell types identified in the granulation tissue. (C) UMAP of the data obtained from the separate tissues involved in analysis. ‘D’ refers to Diseased (granulation tissue), ‘G’ refers to Gingiva (healthy) and ‘L’ refers to periodontal Ligament (healthy) (Cell number per tissue: Diseased granulation tissue = 6729, Gingiva = 4001, Periodontal Ligament = 3024). (D, E) Rate of cell annotation from each tissue resource. (F) Circos plots displaying the number and strength of cell–cell interactions among all cell types across granulation tissue, ligament and gingiva. Node size corresponds to the number of interactions, while edge width indicates the number of significant ligand–receptor pairs between two cell types.

Patients undergoing routine periodontal surgical procedures at the Department of Periodontology, Guy's Hospital, King's College London (KCL), provided consent for the collection of human granulation tissue samples. The study protocol adhered to the guidelines set forth by the UK Human Tissue Act and received ethical approval from the East of England‐Cambridge East Research Ethics Committee (reference 20/EE/0241). Prior to inclusion in the study, written informed consent was obtained from all participants. Inclusion criteria for the patient cohort stipulated the absence of relevant medical conditions, non‐usage of prescribed medication, non‐usage of nicotine or nicotine‐replacement products and non‐pregnancy or breastfeeding status. The granulation samples originated from sites indicated for surgical intervention post provision of step 1 and step 2 of periodontal treatment according the S3 guidelines. All sites included in this research had residual pockets ≥ 6 mm with bleeding on probing post two rounds of non‐surgical periodontal therapy (NSPT) carried out by a specialist trainee at KCL (plaque score < 20%). Post elevation of flap, the remaining granulation tissue attached to the bone was removed using Gracey curettes (Figure 1A). Sample size calculation was done based on sequencing capacity of 10×, to discover new population. Using the platform from Satija Lab (Jeon et al. 2023), it was indicated that, to have at least 10 rare cell types (with 95% confidence), each one presented at a fraction of 3% of the total population containing a minimum of 10 cells from each of these cell types, a minimum of 660 cells needed to be sequenced. Based on our QC showing that each sample was producing an average of 20,000 cells per sample, we know that with *n* = 3 we would achieve a minimum of 6000–10,000 cells sequenced overall, in chips that capture up to 5000 cells.

The granulation samples included in the sequencing are referent from the following surgical sites:

- Patient 1: UR1 intrabony granulation (Male—59 years old—fit and well—generalised periodontitis stage 4 grade C—combination 2–3 wall intrabony defects—surgical intervention carried out < 6 months post last round of NSPT).
- Patient 2: UR6 supracrestal granulation (Female—37 years old—fit and well—generalised periodontitis stage 4 grade C—crater defect without furcation involvement—surgical intervention carried out < 6 months post last round of NSPT).
- Patient 3: LL6 intrabony and supracrestal granulation (Female—30 years old—fit and well‐generalised periodontitis stage 4 grade C—combination 2–3‐wall intrabony defect without furcation involvement—surgical intervention carried out < 6 months post last round of NSPT).

Fresh granulation tissues were dissociated into single‐cell suspension and processed to be sequenced using the Illumina HiSeq 2500 (10× Genomics). Bioinformatic analysis consisted of principal component analysis (PCA), gene ontology (GO), cell–cell communication, single‐cell trajectory analysis and gene‐set enrichment analysis (GSEA). Details can be found in Supporting Information: S1.

## 3 Results

### 3.1 Generation of the Comprehensive Transcriptional Landscape in Disease‐Progressing Granulation Tissue

In this study, we aimed to unravel the intricate transcriptional landscape underlying periodontal granulation tissues by conducting scRNA‐seq analysis and comparing it with healthy gingiva and periodontal ligament, thus allowing us to evaluate the developmental pathway of the cell types inside the control tissues to a disease state (Figure 1A). Starting with an overview of the scRNA‐seq analysis, we integrated our dataset with previously (publicly) available datasets GSE152042 (gingiva) (Caetano et al. 2021) and GSE161267 (periodontal ligament) (Pagella, De Vargas Roditi, et al. 2021). By reprocessing the publicly available raw data and applying stringent quality filtering methods, we obtained single‐cell transcriptomes from a total of 13,754 single cells (granulation tissue = 6729, gingiva = 4001, periodontal ligament = 3024). Unbiased clustering of the cells identified 10 clusters based on uniform manifold approximation and projection (UMAP) analyses, and the cell types were annotated according to previously published datasets (Caetano et al. 2021; Pagella, De Vargas Roditi, et al. 2021) (Figures 1B,C, S1 and S2). We investigated the proportion of each cell cluster, comparing between the sample sets (Figure 1D,E). Our results identified that diseased granulation tissue had a higher abundance of immune/plasma cells (69.5% of the total cell count) compared to healthy gingiva and PDL (23.7% and 14.8%, respectively). KEGG pathway enrichment analysis showed that the immune compartment was enriched for osteoclast differentiation pathways (Figure S3). We also identified a small population of epithelial cells (3.3%), pericytes (5.5%) and endothelial cells (11%) in the granulation tissues. Interestingly, the proportion of fibroblasts in granulation tissue was not significantly different from that in healthy tissues. These findings deepen our understanding of the cellular composition and molecular signatures associated with periodontal granulation tissues.

### 3.2 In Silico Dissection of the Granulation Tissue

To investigate the role of the different cell compartments in the granulation tissue, we performed UMAP subcluster analysis. To understand which populations we should focus on, we first conducted a cell–cell communication analysis using the CellChat package on the collective of these three tissues (Figures 1F and S4). Our results showed that the populations producing the largest weight of interactions were the fibroblasts, endothelial cells, epithelial cells and pericytes (parenchymal tissue). Additionally, our cell–cell communication analysis also highlighted that both endothelial and fibroblast cell clusters showed the most pronounced changes in signalling pathways. Specifically, endothelial cells are shown to receive signals, while fibroblasts are responsible for their release. This interaction underscores the critical roles these two cell types play in mediating communication and functional responses within the tissue microenvironment.

Although the immune compartment did not show relevance in the cell communication analysis, the changes in cell number abundance from health to disease led us to characterise their sub‐compartments (Appendix A, Figures S5–S9). In contrast to parenchymal tissues—whose constituent cell types are stably present in gingival and PDL structures—the immune compartment is transient and must be enriched by isolating CD45^+^ cells for sequencing (Chen et al. 2022; Liu et al. 2023), which limited our ability to perform lineage‐commitment analyses. Therefore, because CellChat analysis results showed that communication interactions were more prevalent in the cell types from the parenchymal compartment (Figure 1F), we proceeded with subcluster analysis focused on these populations to understand these cell lineage changes from health to disease.

### 3.3 Subcluster Analysis of Epithelial Cells

**FIGURE 2:**
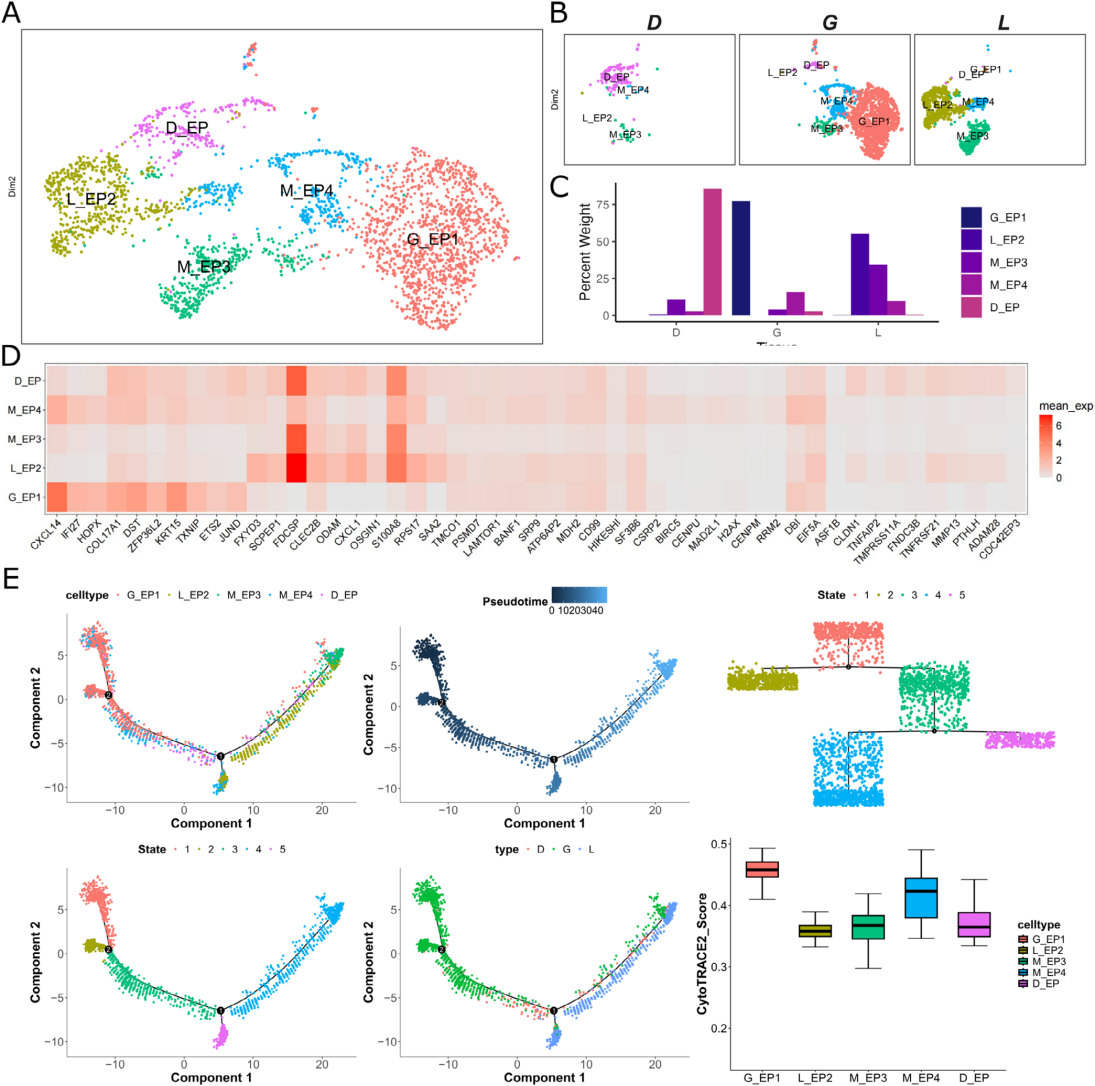
Epithelial subpopulation analysis. (A) UMAP plot showing reclustering of epithelial cells, with individual cells coloured by cluster annotation. (B) UMAP representation of epithelial cells, differentiated by tissue origin. (C) Rate of cell annotation from each tissue resource. (D) Heatmap displaying differentially expressed genes (DEGs) among epithelial cell subclusters, ranked by log2‐fold change (log2FC). (E) Trajectory analysis revealing differentiation relationships among epithelial cells. Differentiation starting points were identified using CytoTRACE2, and pseudotime trajectories were inferred using Monocle2. Trajectory plots were visualised by pseudotime, tissue origin, cell type and differentiation stage.

The proportion of epithelial cells in granulation tissue is significantly lower than in the gingiva and ligament (Figure 1E). Inside this small epithelial compartment, five subclusters were identified, of which two subclusters were of mixed origin (M_EP3 and M_EP4) and the other three were specific to each tissue: granulation (D_EP), gingiva (G_EP1) and PDL (L_EP1) (Figure 2). Gene ontology (GO) analysis of the populations revealed distinct enrichment for each subpopulation, with D_EP enriching for wound healing and regulation of cell projection adhesion (Figure S10). This subset showed up‐regulation of differentiation markers such as *KRT13* and *CLDN1*, along with the down‐regulation of basal markers *KRT14* and *KRT15*, indicating a transition from progenitor activity towards differentiation to restore the junctional epithelial barrier (Figure S11) (Fujita et al. 2010). Additionally, the D_EP subpopulation expressed higher levels of vimentin (*VIM*), indicating a potential epithelial‐to‐mesenchymal transition (EMT), which might facilitate bacterial invasion into the underlying gingival tissues and propagation of inflammation (Saliem et al. 2022). To understand the potency of differentiation capacity and the trajectory of differentiation among the epithelial populations, we employed pseudotime and CytoTRACE2 analyses. These analyses revealed that D_EP was highly differentiated compared to G_EP1, suggesting that the granulation tissue epithelial cells possess low developmental potential (Figure 2E). Furthermore, the trajectory places M_EP4 immediately upstream of D_EP, indicating that M_EP4 cells give rise to this most differentiated population.

### 3.4 Subcluster Analysis of Pericytes

**FIGURE 3:**
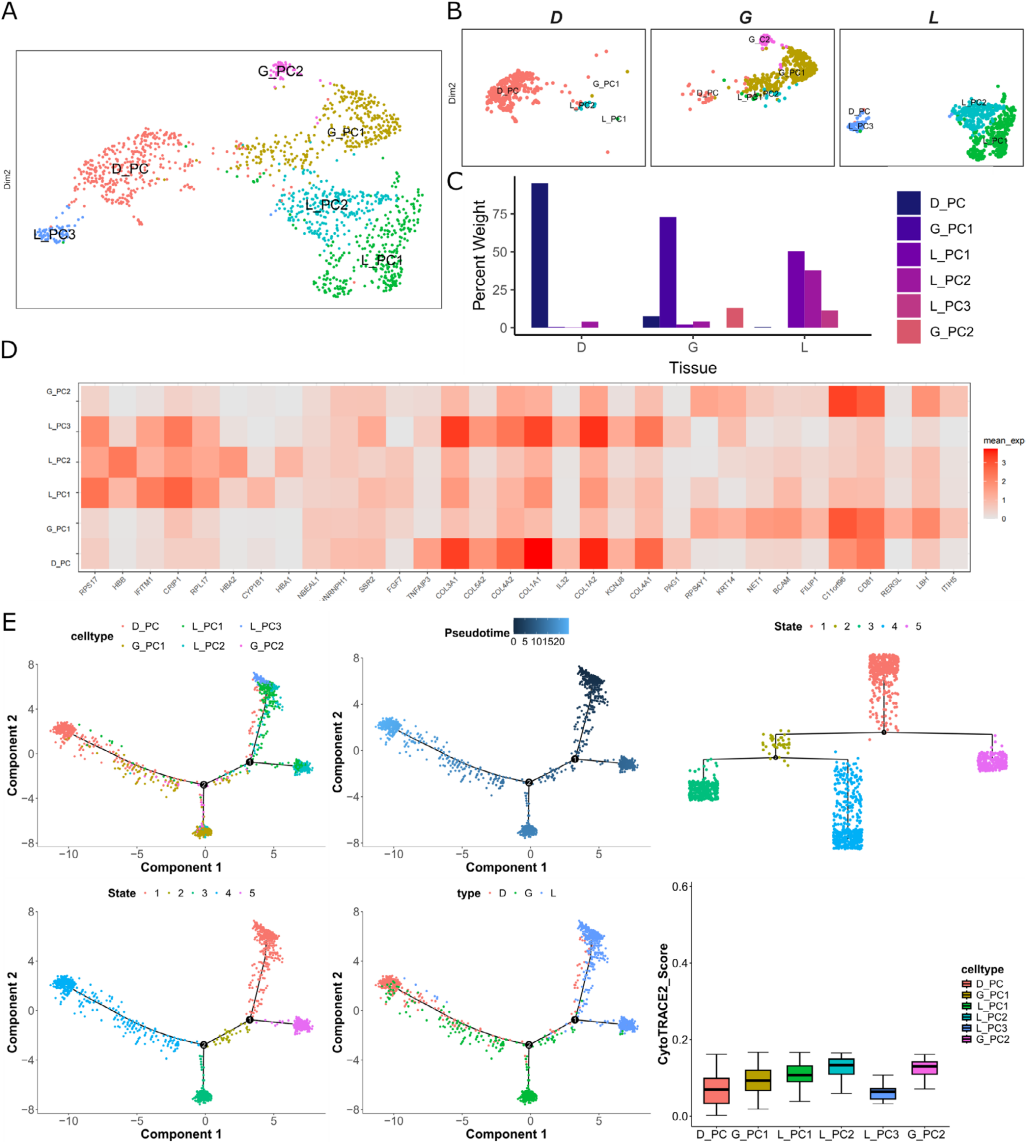
Pericytes subpopulation analysis. (A) UMAP plot showing the reclustering of pericytes, with individual cells coloured by cluster annotation. (B) UMAP representation of pericytes differentiated by tissue origin. (C) Rate of cell annotation from each tissue resource. (D) Heatmap displaying differentially expressed genes (DEGs) among pericyte subclusters, ranked by log2‐fold change (log2FC). (E) Trajectory analysis revealing differentiation relationships among pericytes. Differentiation starting points were identified using CytoTRACE2, and pseudotime trajectories were inferred using Monocle2. Trajectory plots are displayed based on pseudotime, tissue origin, cell type and differentiation stage.

Within the pericyte compartment, six subclusters were identified, of which one was specific to granulation tissue (D_PC) (Figure 3). GO analysis of the populations revealed that D_PC was enriched for small GTPase‐mediated signal transduction and amoeboid‐type cell migration, suggesting that this subcluster plays a pivotal role in the dynamic cellular processes involved in tissue remodelling and repair (Figure S12). The enrichment for small GTPase‐mediated signal transduction indicates a regulatory function in cytoskeletal organisation (El Masri and Delon 2021), while the association with amoeboid‐type cell migration suggests a capacity for rapid and adaptive movement through the extracellular matrix (ECM) (Yamada and Sixt 2019), facilitating granulation tissue formation. These findings highlight the specialised functions of D_PC within the granulation tissue microenvironment. Pseudotime and CytoTRACE2 analyses revealed that D_PC was highly differentiated in comparison to PDL and gingival PC populations, with the cells from the G_PC1 population comprising the majority on the trajectory of becoming D_PC, and a small proportion of L_PC1 on the same trajectory (Figure 3E). Altogether, these findings suggested that a small subpopulation of gingival and PDL cells takes on a differentiation commitment into the D_PC population and that the D_PC population displays a distinct functional trajectory to the gingiva and PDL pericytes.

### 3.5 Subcluster Analysis of the Fibroblasts

**FIGURE 4:**
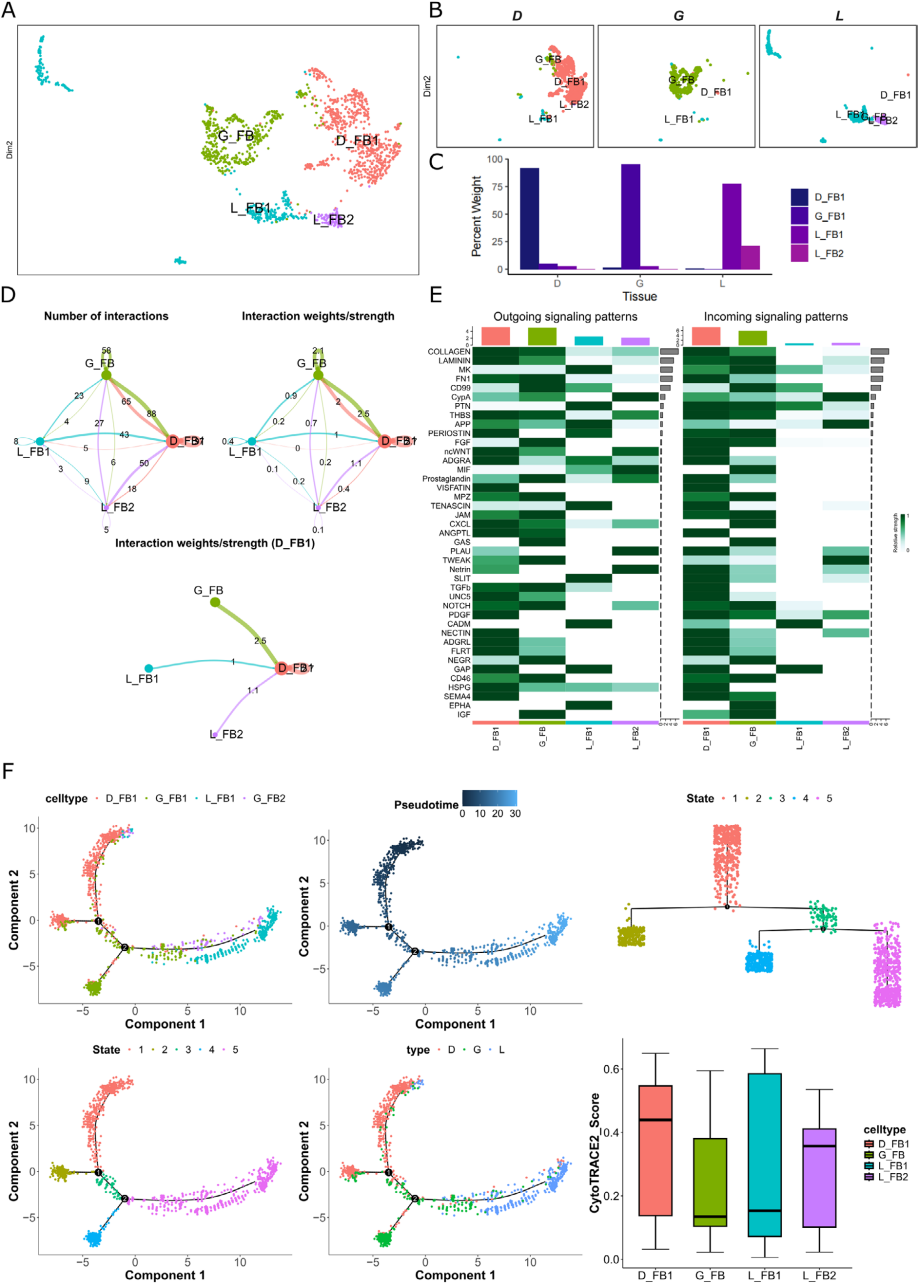
Fibroblast subpopulation analysis. (A) Reclustering UMAP plot of fibroblast cells, single cells coloured by cluster annotation. (B) UMAP plot divided by tissue. (C) Rate of cell annotation from each subpopulation. (D) Circos plot depicting cell interactions among the four cell subtypes within the fibroblast populations. (E) Signalling sender and receiver information and enriched as clusters. (F) Pseudotime plots revealing the differentiation trajectory of fibroblasts cells, annotated by cell type, state, cluster and tissue source, and CytoTrace2 identification of the starting point of fibroblast cell differentiation.

The fibroblast subclustering analysis revealed four subclusters, with the granulation subcluster (D_FB1) being enriched in the functional GO analysis for ECM organisation, extracellular structure organisation and external encapsulating structure organisation, suggesting that D_FB1 plays a critical role in shaping the ECM architecture during granulation tissue formation (Figures 4 and S13). Cell‐to‐cell interaction analysis of the fibroblast subcluster revealed that D_FB1 had the largest weight of interaction of outgoing and incoming signalling patterns, singling and receiving Collagen, Laminin, Midkine and Fibronectin pathways, all of which could be therapeutic targets (Figure 4D,E). Pseudotime and CytoTRACE2 analyses revealed that D_FB1 was highly undifferentiated in comparison to PDL and gingival populations (Figure 4F), suggesting that D_FB1 represents a progenitor‐like fibroblast population that is poised to respond dynamically to reparative cues in the granulation tissue microenvironment. Additionally, D_FB1 cells uniquely expressed *LEF1*, a key transcription factor in the Wnt signalling pathway that regulates cell proliferation and differentiation (Liu and Millar 2010; Clevers 2006), and increased expression of *HSPA1A* and *HSPA1B*, which are genes associated with metastatic colon cancer (Guan et al. 2021) (Figure S14). Notably, D_FB1 displays elevated levels of other cancer‐ and inflammation‐related genes—such as *DNAJB1*, *ZFP36*, *JUND*, *TNFAIP3* and *IL6*—further underscoring a microenvironment characterised by heightened cellular stress and chronic inflammation (Kim and Hong 2022; Carrick et al. 2004; Zhou et al. 2023; Ma and Malynn 2012; Kumari et al. 2016). In particular, IL6 is a proinflammatory cytokine frequently implicated in tumour progression, metastasis and periodontitis (Kumari et al. 2016; Balta et al. 2021) (Figure S14). Together, these data support the idea that the fibroblasts in this tissue are more inclined towards a low‐differentiation state but with a unique profile that indicates that D_FB1 could be a critical population orchestrating the progression and formation of the phenotype seen in the granulation tissue.

### 3.6 Subcluster Analysis of Endothelial Cells

**FIGURE 5:**
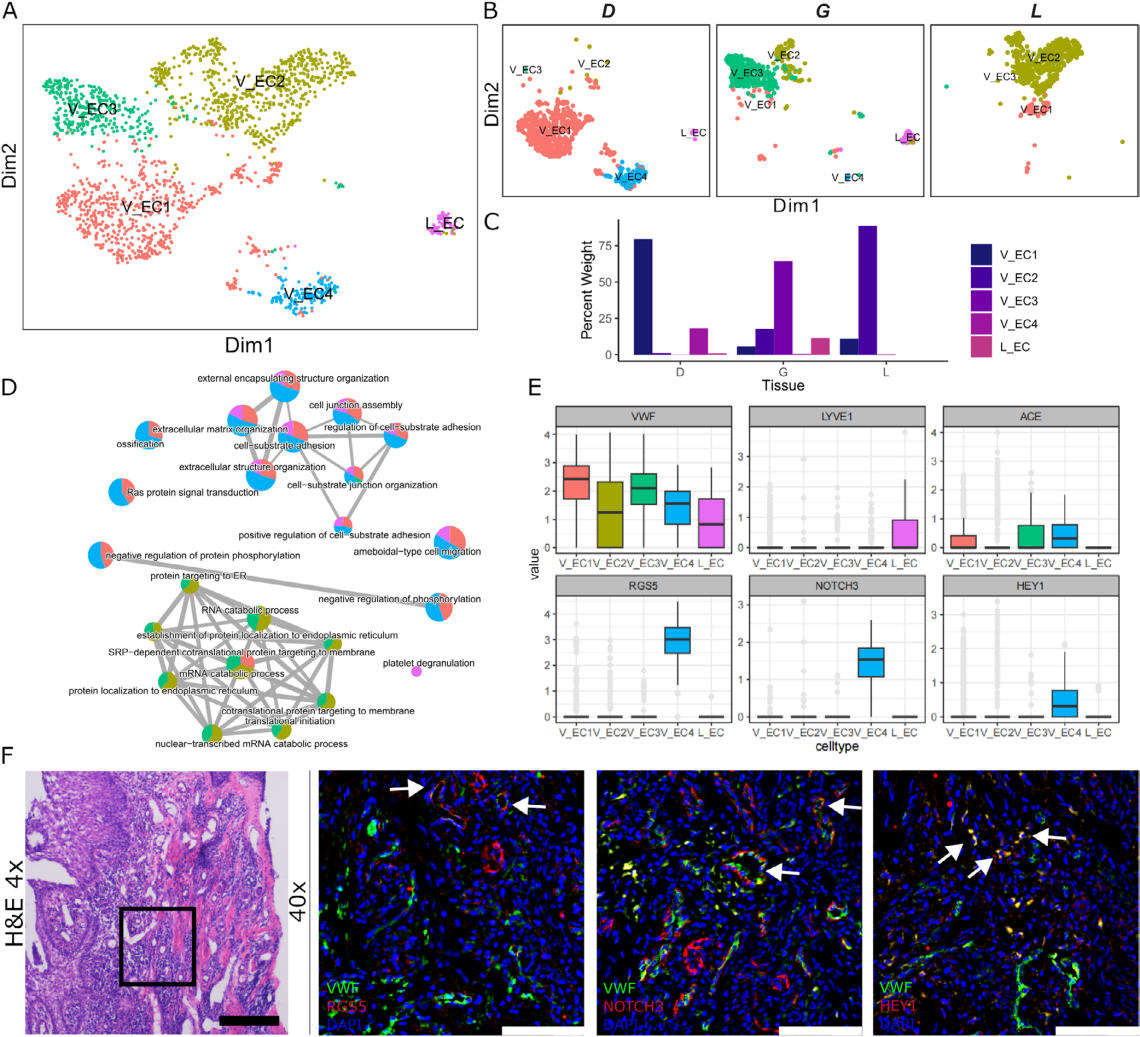
Endothelial subpopulation analysis. (A) Reclustering UMAP plot of endothelial cells, single cells coloured by cluster annotation. (B) UMAP plot divided by tissue. (C) Rate of cell annotation from each subpopulation. (D) GO enrichment terms for comparing clusters. (E) Box plot showing subset‐specific markers and their expression levels within the endothelial subclusters. (F) Validations and mapping of unbiasedly identified populations based on the expression of selected marker genes. White arrows showing co‐localisation; Scale bars: 4×, 500 μm, 40×, 100 μm.

Finally, to identify the role of endothelial cells in the periodontal tissues we performed subcluster analysis, which revealed the presence of five different subclusters, among which two were specific to granulation tissue (V_EC1 and V_EC4) (Figure 5A–C). GO enrichment analyses revealed a pronounced involvement of ossification pathways in the endothelial cell compartments V_EC1 and V_EC4 (Figures 5D and S15A,B), particularly in cluster V_EC4, which originates specifically from granulation tissue (Figure 3D,E). To characterise the vascular endothelium, we created a marker gene plot that showed von Willebrand factor (*VWF*) expression in all clusters, establishing its identity as a vascular endothelial marker (V_EC) (Figure 5E). Conversely, *LYVE1*, indicative of the lymphatic endothelium (Li et al. 2021), was exclusively detected in cluster L_EC, originating mainly from gingiva. To validate the presence of the V_EC4 subcluster in the granulation tissue, we identified on the granulation tissues *VWF+* co‐location with *RGS5*, *NOTCH3* and *HEY1*, as predicted by the marker gene plot (Figure 5F), confirming that this new population of cells exists in situ.

**FIGURE 6:**
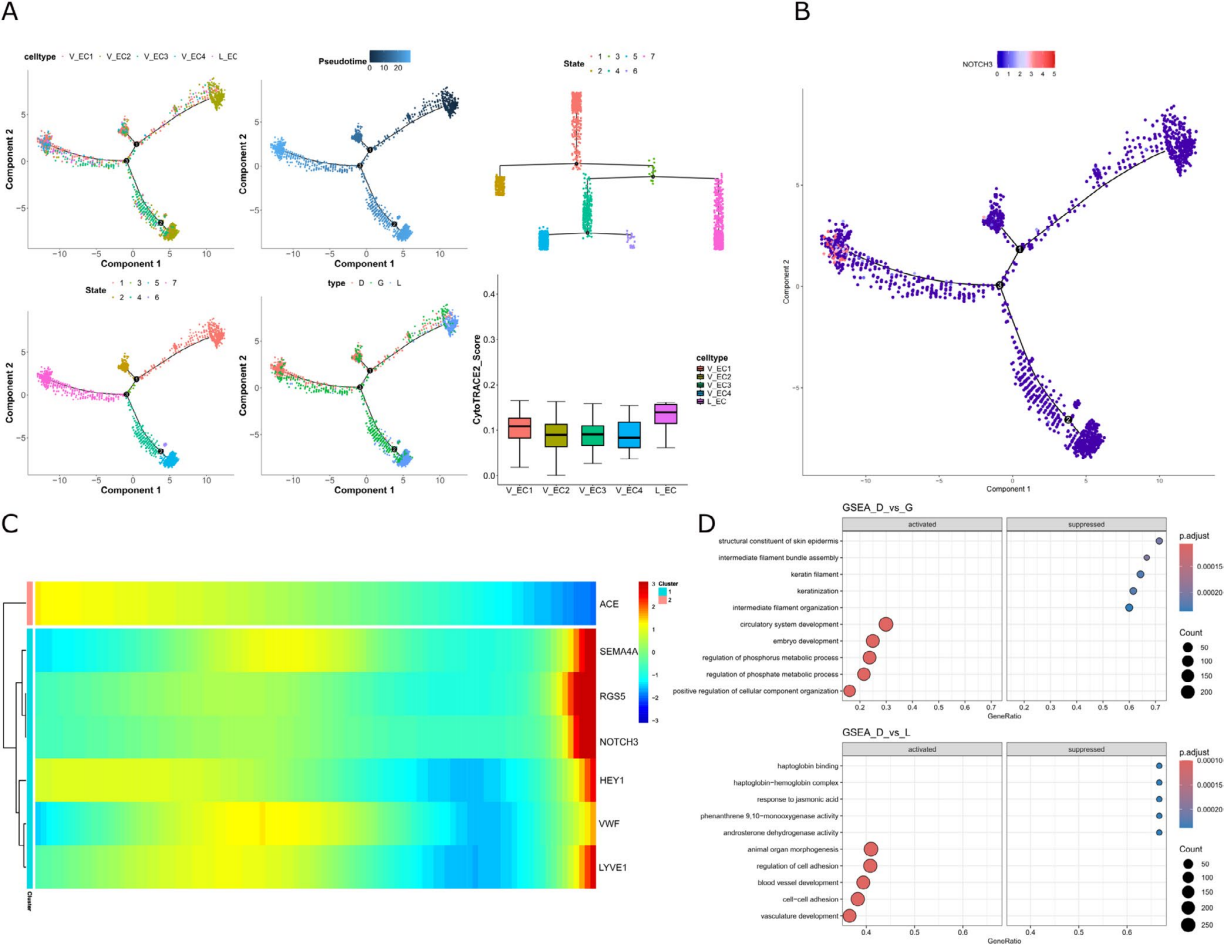
Pseudotime analysis elucidating the origin of endothelial cells in periodontal tissues. (A) Pseudotime plot revealing the differentiation trajectory of endothelial cells, annotated by cell type, state, cluster and tissue source. CytoTrace2 identifies the starting point of endothelial cell differentiation. (B) Pseudotime plot showing high NOTCH3‐specific expression where granulation‐specific population V_EC4 is located. (C) Marker genes for endothelial cell identification plotted along pseudotime, illustrating their dynamic expression profiles during differentiation. These genes are further clustered based on their expression patterns. (D) Gene‐set enrichment analysis (GSEA) identifying enriched pathways among different tissue resources. This comprehensive analysis provides insights into the developmental trajectory and functional characteristics of endothelial cells in periodontal tissues.

To explore cluster‐specific functions and track the developmental origin of cell types, pseudotime analysis was performed using Monocle2, and the starting point of differentiation was determined by CytoTRACE2 (Figure 6A–C). While lymphatic endothelial (L_EC) cells exhibited the lowest degree of differentiation, the granulation‐specific V_EC4 subpopulation, characterised by *NOTCH3* expression and its involvement in ossification, demonstrated the highest degree of differentiation. According to the pseudotime trajectory, V_EC4 emerges along the branch that extends from V_EC3, prevenient from the gingival tissues and is characterised by *VWF* and *ACE* expression (Figure 5D). Subsequently, we conducted GSEA comparing the disease tissue with gingiva and PDL (Figure 6D). The most significantly enriched pathways emphasised vascular development–related pathways (Figure S15C).

**FIGURE 7:**
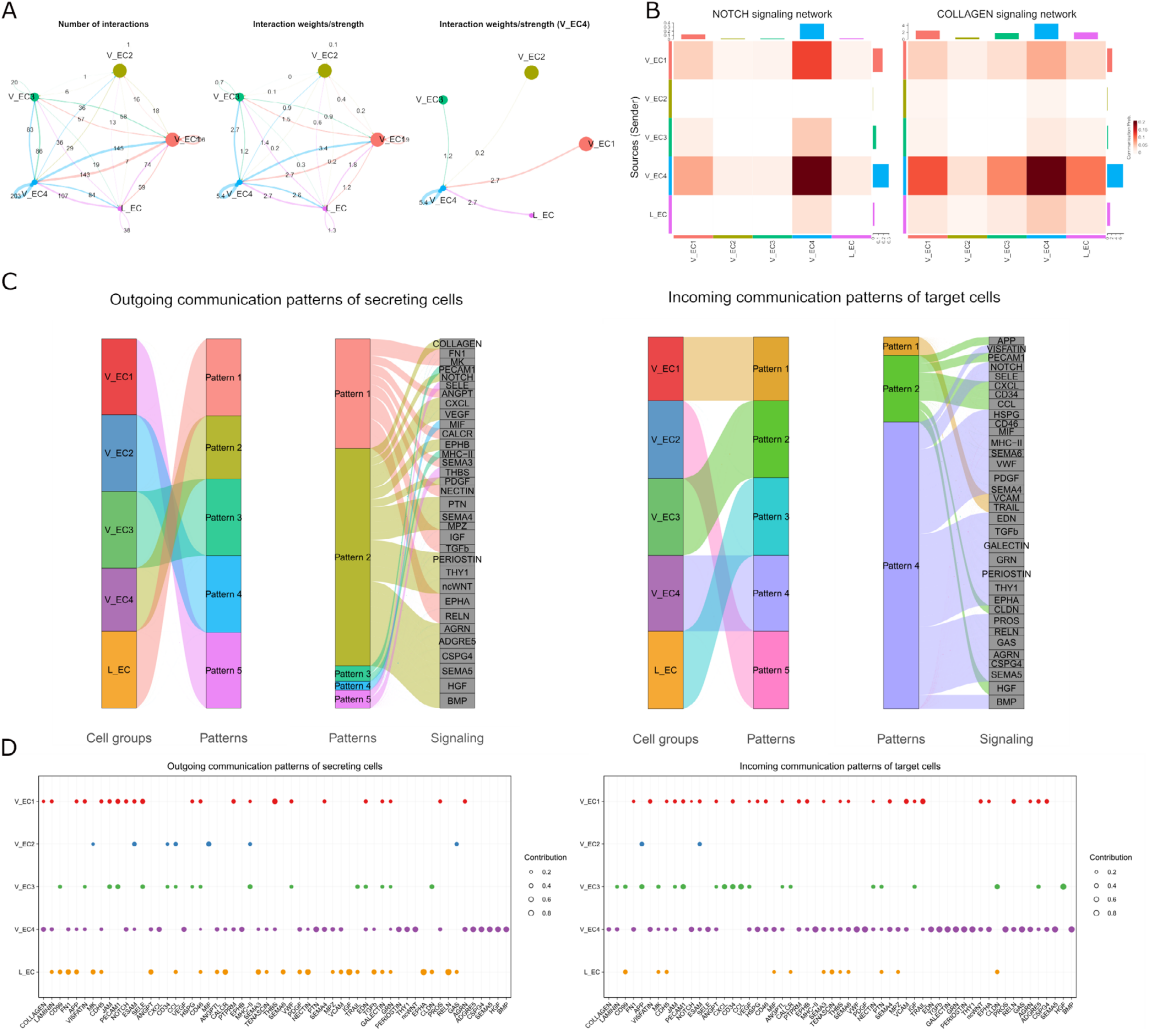
Cell–cell communication among endothelial types in the periodontal pathogenesis microenvironment. (A) Circos plots displaying the number and strength of cell–cell interactions among all endothelial cell subtypes across granulation tissue, ligament and gingiva. (B) Heatmaps summarising specific signals between interacting cell types. Interactions are divided into outgoing and incoming events for specific cell types. The colour gradient indicates the relative strength of the interactions. (C) Signalling sender and receiver information is presented on the riverplot and enriched as clusters. (D) Dot plot illustrating the altered signalling pathways among each subtype of endothelial cell. V_EC4 has enriched the most significant outgoing and incoming signalling.

Given the potential functionality discovered of the endothelial compartment of the granulation tissue, we employed a cell‐to‐cell communication analysis to investigate the mechanism by which these populations regulated the environment. Our analysis revealed that the granulation tissue–specific clusters (V_EC1 and V_EC4) displayed the largest weight of interaction, with the V_EC4 cluster having the most robust signalling enrichment of all endothelial clusters (Figure 7A), suggesting that V_EC4 plays a crucial role in the diseased periodontal microenvironment. This population signalling is significantly marked by the NOTCH pathway, the interaction analysis of which revealed that V_EC4 interacted with the VEGF, COLLAGEN and BMP pathways, all crucial for vascular and ossification development (Figures 7B–D and S16). Our analysis suggests that the V_EC4 endothelial population is crucial for both vascular development and ossification of the microenvironment of periodontal disease.

## 4 Discussion

Using scRNAseq, we obtained high‐resolution transcriptomic data from a diverse range of cell populations present in the periodontal microenvironment from healthy tissues to the development of granulation tissue, the sequelae consequent of the disease. Our findings shed light on the molecular complexity of this tissue, allowing us to characterise the cell populations within the granulation tissues, as well as understanding the trajectory of differentiation from healthy to disease, providing valuable insights into the pathogenesis progression of periodontal disease, and unveiling important insight into a distinct endothelial cell subpopulation (V_EC4) in granulation tissue, characterised by the NOTCH pathway signalling.

The endothelial‐derived NOTCH pathway expression is extensively documented as promoting angiogenesis and osteogenesis in bone (Ramasamy et al. 2014). Our new population was found specifically in the diseased endothelial compartment, expressing *NOTCH3*. A unique aspect of *NOTCH3*, in comparison to the other members of the Notch family, is its restricted pattern of tissue distribution, found in vascular cells, the central nervous system and subsets of thymocytes (Aburjania et al. 2018). Although the deletion of *Notch1* and *Notch2* is lethal to a developing embryo, deletion of *Notch3* is not (Hamada et al. 1999; Krebs et al. 2003); nevertheless, Notch3‐null mice show decreased development of normal vasculature, highlighting that abnormalities in this protein are linked primarily to vascular pathologies (Joutel et al. 2010). Further, *Notch3*+ populations have been described as a stem cell source for novel odontoblast formation in dentine damage (Pagella, Roditi, et al. 2021), highlighting its potential to be a source of stem cells in this environment. Additionally, *NOTCH*+ endothelial cells were co‐localised to *RGS5* and *HEY1*. RGS5 has been described in the dental pulp as a source of stem cells to produce odontoblasts (Krivanek et al. 2020). Hey1 is a downstream effector of the canonical Notch signalling (Buas et al. 2010) and TGF‐b/BMP signalling, independently of Notch (Wöltje et al. 2015). Our results suggest that this population plays a crucial potential role in shaping the diseased microenvironment, emphasising the close association between vascular development and ossification processes. Further functional analysis is needed to confirm these results; however, targeting this population would be a potential, novel therapeutic target for enhancing periodontal regeneration.

Our findings also underscore the dynamic interplay between various cell types within the periodontal disease progression‐related tissue, highlighting that fibroblasts are the most interactive cell type during disease progression. Fibroblasts, particularly the D_FB1 subcluster, exhibit a progenitor‐like state with pronounced ECM organisation activity. This population's low differentiation state and its association with the Wnt signalling pathway suggest a crucial role in the granulation phenotype observed in periodontal disease. A biologically similar event takes place with cancer‐associated fibroblasts (CAFs), where they have the potency to differentiate into a functional fibroblast that produces ECM structures and metabolic and immune reprogramming of the tumour microenvironment impacting tumour progression (Yang et al. 2023). Therefore, further biological characterisation of this population in relation to cancer samples is needed to confirm the findings as well as to understand how they play a role in the granulation tissue phenotype. Ultimately, modulation of this fibroblast population's expression in the granulation tissue could be crucial for managing the progression of periodontal disease.

Recent studies using scRNAseq and advanced molecular profiling have similarly highlighted the importance of cellular heterogeneity and signalling networks in periodontal tissue homeostasis and disease progression. Our research's uniqueness stems from the fact that the collection of tissue was performed following flap elevation, harvesting via curettage the granulation tissue attached to the bony walls, whereas the majority of the scRNAseq was performed in resected gingival tissues (Caetano et al. 2021). One of the limitations of our study is the fact that we used previously published datasets, introducing potential variability in sample collection methods and demographics, which could influence molecular profiles. To mitigate this, we reprocessed the raw sequencing files using the same genome reference and standardised the analysis pipeline (via Cell Ranger) to minimise batch effects. Additionally, the granulation tissue analysed in this study reflects a post‐treatment state (following two rounds of subgingival instrumentation), and further studies are needed to understand whether the granulation tissues microenvironment change before and after treatment. Nevertheless, the tissues collected for this study came from residual pockets (≥ 6 mm with BOP); therefore, these sites represent active, unresolved periodontal disease.

Notably, our findings align with previous studies showing significant cell‐type shifts in periodontal disease highlighted by the invasion of immune cells (Caetano et al. 2021; Chen et al. 2022; Liu et al. 2023; Zitzmann et al. 2005) and hyperactive inflammatory markers. Here we showed that this invasion is mainly present in the granulation tissue and could be the promoter of the fibroblast phenotype we identified. Additionally, multiple stem/progenitor populations in the gingiva and PDL were identified with the role of maintaining tissue integrity and modulating repair responses (Caetano et al. 2021; Yang et al. 2024; Pagella, De Vargas Roditi, et al. 2021). In line with those findings, we observed the end of the line of the differentiation of these progenitor cells, identifying their lineage commitment towards the diseased granulation tissue and their function, demonstrating their potential influence either towards periodontal regeneration or disease progression outcomes. Together, this confluence of evidence positions our study within a broader literature demonstrating that single‐cell transcriptomic approaches can uncover previously unappreciated heterogeneity in cell populations driving both the destructive and reparative processes of periodontal disease.

Clinically, there is debate as to whether the granulation tissues should be left or removed. Our dataset shows that the microenvironment of the granulation tissue is intricate, with some supporting removal of the tissue (D_FB1) and some suggesting maintaining it (V_EC4). Nevertheless, current clinically available host modulators used in periodontal disease management are not based either on targeting these cell populations nor on the pathways presented in our research (Donos et al. 2020). Therefore, there is scope for developing and exploring novel host modulators that target the populations and pathways involved in periodontal disease progression or regeneration (e.g., Notch pathway), advancing therefore periodontal disease management.

In conclusion, our study advances the current understanding of the pathogenesis of periodontal disease by characterising the most important regulatory compartments of the granulation tissue and understanding their developmental origins in relation to periodontal healthy tissues.

## Author Contributions

V.C.M.N. conceptualised, designed and performed experiments; curated and analysed the data; and wrote, edited and reviewed the original manuscript. W.Z. performed the bioinformatic analysis and wrote, edited and reviewed the original manuscript. K.F. and P.D. performed the single‐cell RNAsq. J.K. critically revised the manuscript. C.Z., P.S. and L.N. provided the resources and reviewed the manuscript. All authors reviewed the manuscript.

## Conflicts of Interest

The authors declare no conflicts of interest.

## Supporting information


**Data S1:** jcpe70048‐sup‐0001‐supinfo.docx.

## Data Availability

All data associated with this study is present in the paper itself or in the Supporting Information. Requests for data should be addressed to the corresponding authors. Raw sequencing data obtained from patients used in this study is deposited in the National Center for Biotechnology Information accession GSE294615.

## Appendix A

### Subcluster Analysis of Immune Cell Compartments

We identified five major subcluster types across periodontal samples: macrophages, mast cells, memory B cells, plasma cells and T cells. Among these, plasma cells were notably enriched in granulation tissue, increasing 13 times the number of cells in comparison to healthy tissues, revealing robust up‐regulation of *IGHG1*, *IGHG3*, *IGLC3* and *CD52* populations, suggesting a localised humoral immune response in chronic inflammation (Figure S5). The memory B‐cell subcluster also showed a 5.5 times increase in cell numbers in the diseased tissues with significant increase of *MS4A1* (*CD20*) expression in one of the populations (Figure S6). Mast cell subpopulation analysis demonstrated an enrichment of a population expressing immunoglobulin‐related transcripts *IGKC* and *IGLC2* in the diseased tissue (Figure S7). T cells were moderately abundant across all tissues but lacked subset‐specific markers, suggesting steady‐state or transitional activity rather than distinct effector subsets (Figure S8). Finally, macrophage subpopulation characterisation did not reveal a clear shift in M1/M2 polarisation. Nevertheless, our analysis highlighted an increase in populations expressing *TSPAN13*, *CLEC9A*, *S100A8* and *S100A9*, suggesting osteoclastic differentiation and pro‐inflammatory activity participation (Figure S9).

Parenchymal cells (fibroblasts, endothelial cells and other stromal populations) are bona fide tissue residents whose differentiation continua were well captured by our whole‐tissue approach; however, the immune compartment is transient. For appropriately capturing this population, isolating the CD45^+^ cells (Kinane et al. 2017; Van Dyke et al. 2020) is necessary prior to sequencing. Therefore, we could not reliably distinguish long‐lived tissue‐resident immune cells from transient blood‐derived infiltrates.
