# Supplementary material for: Characterising the Periodontal Granulation Tissue Using scRNAseq
Source: J Clin Periodontol. 2025 Oct 7;53(2):308–20. doi: 10.1111/jcpe.70048 (PMC12803696; doi:10.1111/jcpe.70048)
Supplement: Supplementary file 1 — Data S1: jcpe70048‐sup‐0001‐supinfo.docx. [file JCPE-53-308-s001.docx]

**Supplementary Figures**

**
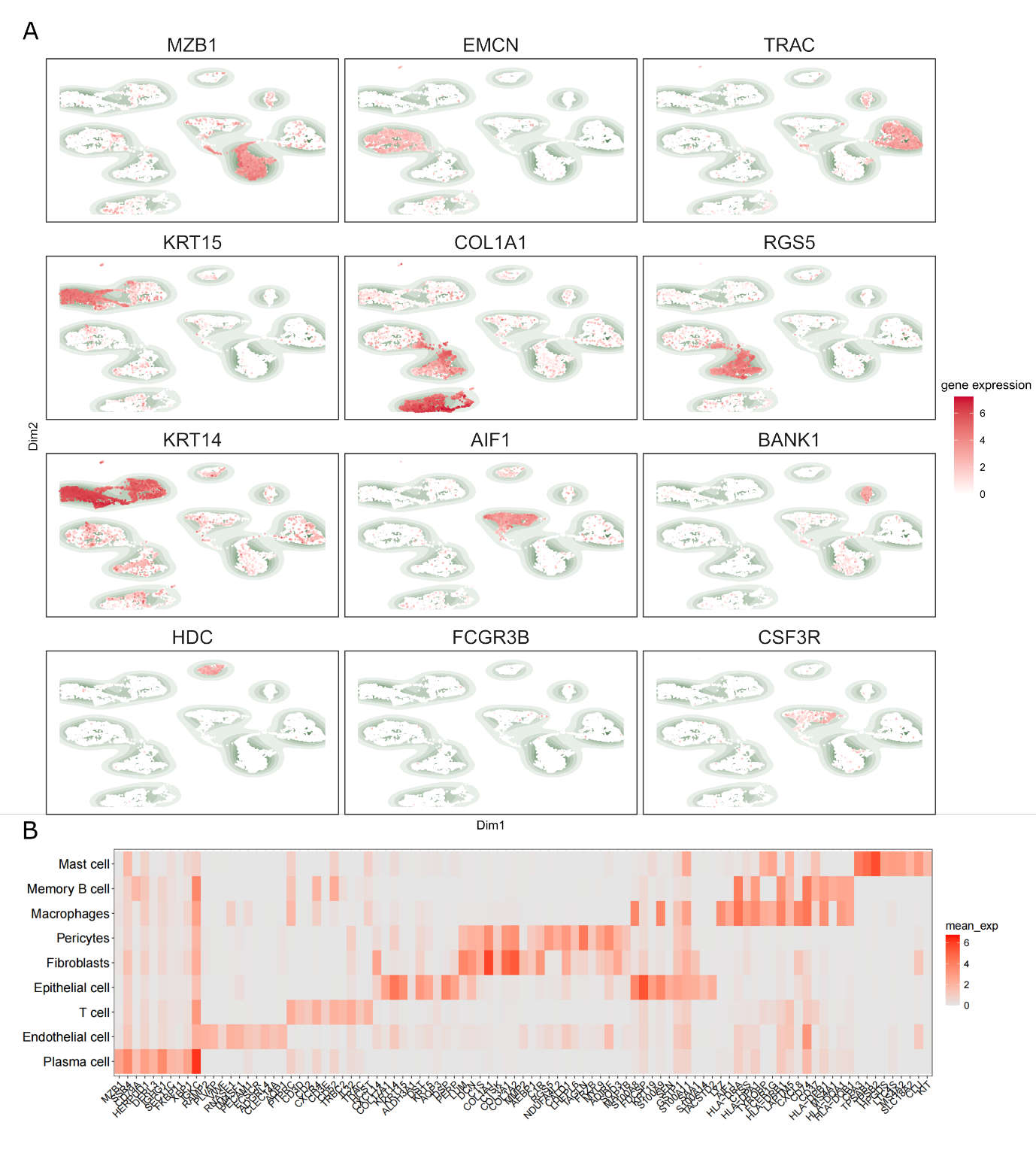
Supplementary Figure 1. Clustering marker genes**

(A) Selected markers used for cell type identification correlating to the UMAP of Figure 1. (B) Differentially expressed genes (DEGs) were normalized and visualized on a heatmap, displaying the Top 10 genes ranked by log2 fold change (log2FC).

**
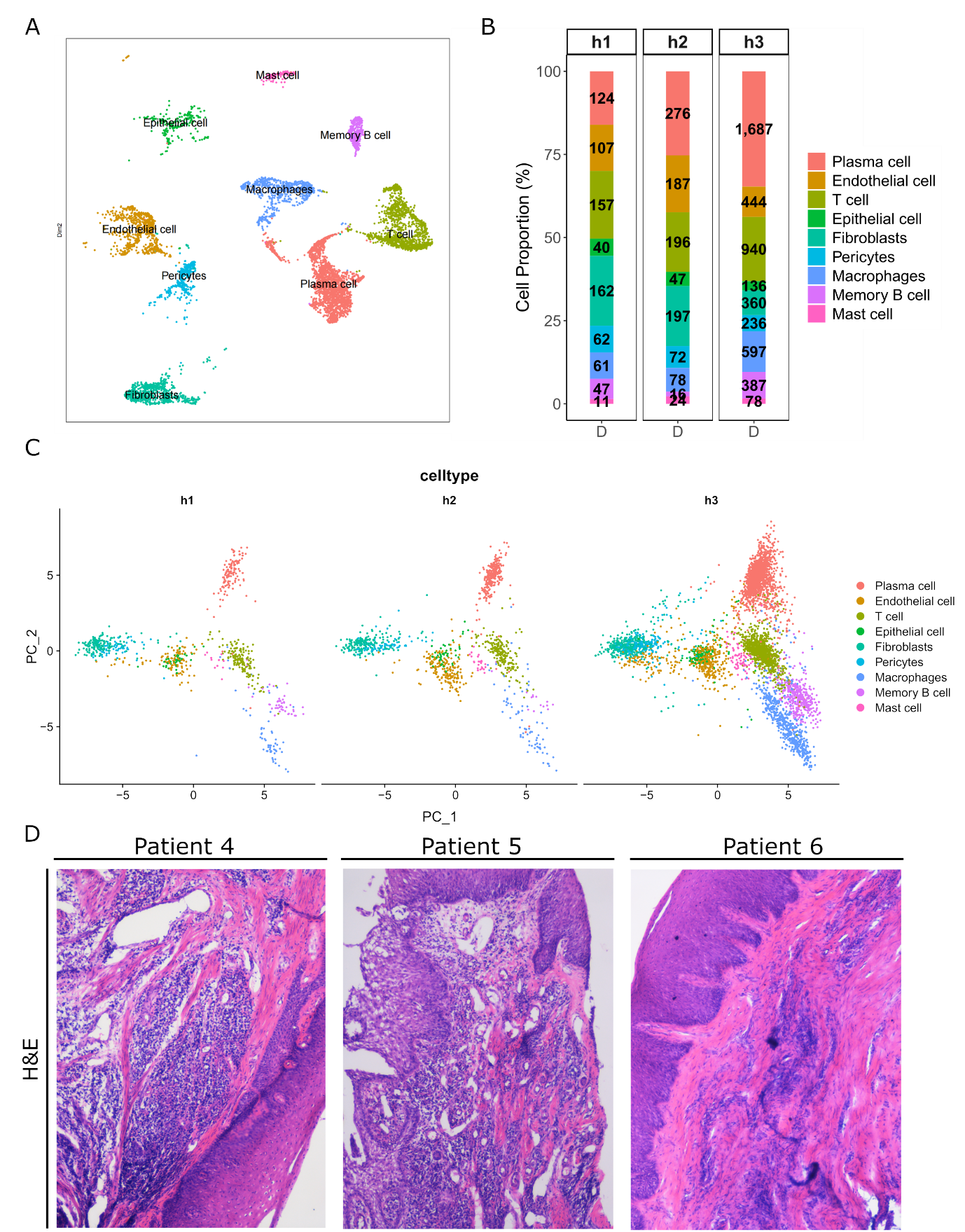
**

**Supplementary Figure 2. Overview of single cells from granulation tissue samples**

(A) Uniform Manifold Approximation and Projection (UMAP) representation showing nine cell types identified in the granulation tissue. (B) Cell proportion analysis displaying cell number per individual sample used in the sequencing analysis (h1 corresponds to patient 1, h2 h1 corresponds to patient 2, and h3 corresponds to patient 3). (C) PCA plot of individual samples shows homogeneity of cellular populations across samples. (D) Haematoxylin and eosin (HE) staining of biopsies from granulation tissue from different patients from those that were sequenced, from samples collected during gingival resection – we used this method to have orientation on doing histology.


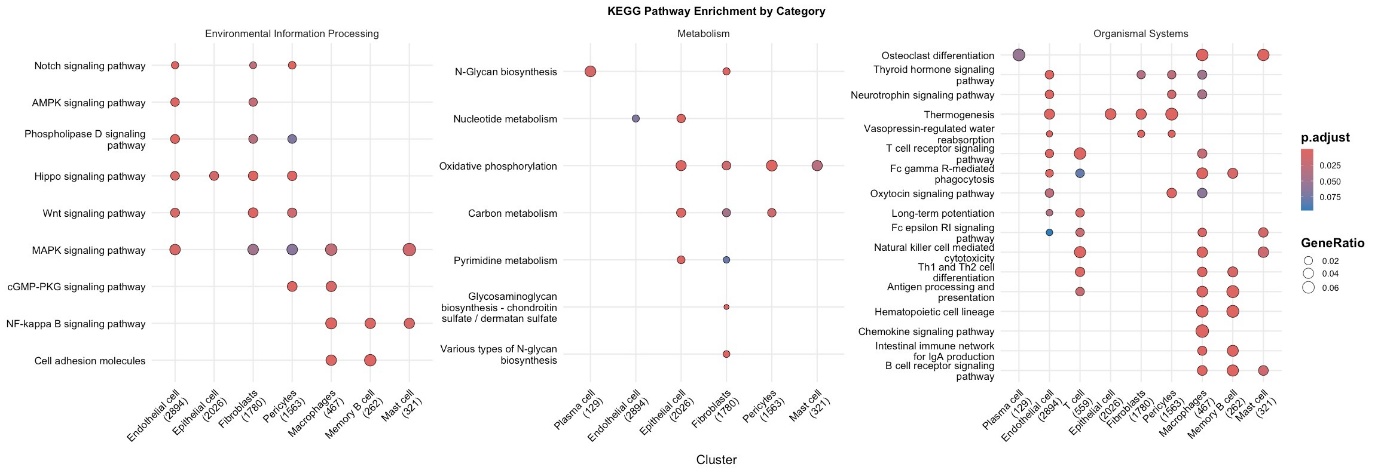


**Supplementary Figure 3. KEGG Pathway Enrichment Analysis of Granulation Tissue Cell Types**

Within the granulation tissue, KEGG pathways enriched using and shown by dotplot from the function of clusterProfiler, categorized by pathway type and split by cell types. Pathways are ranked by adjusted p-value (P.adjust), with a significance threshold set at pvalueCutoff = 0.05.


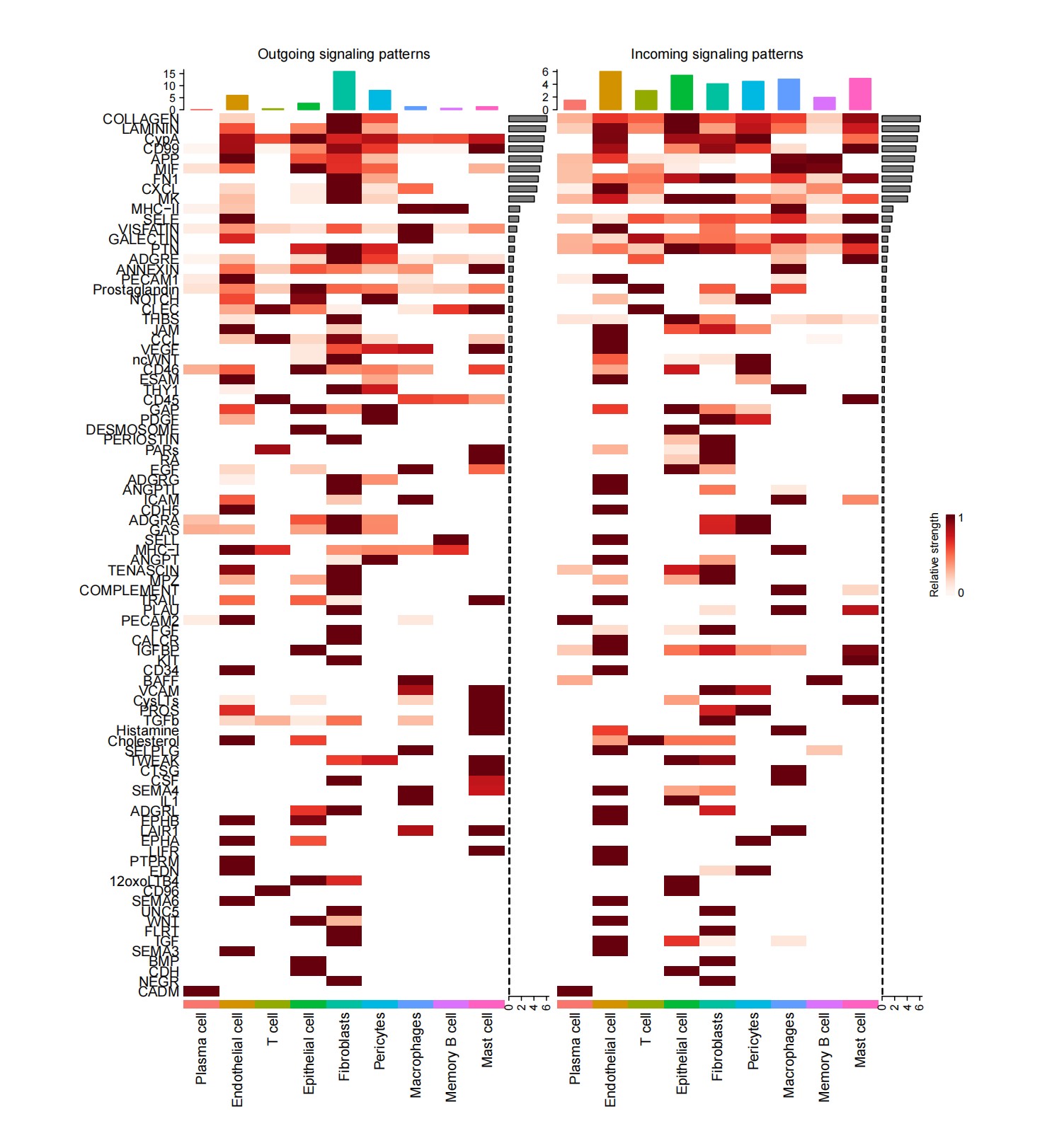


**Supplementary Figure 4. Cell-Cell Interaction Dynamics Across Tissues**

Heatmaps summarizing specific signals between interacting cell types. Interactions are divided into outgoing and incoming events for specific cell types. The colour gradient indicates the relative strength of the interactions.


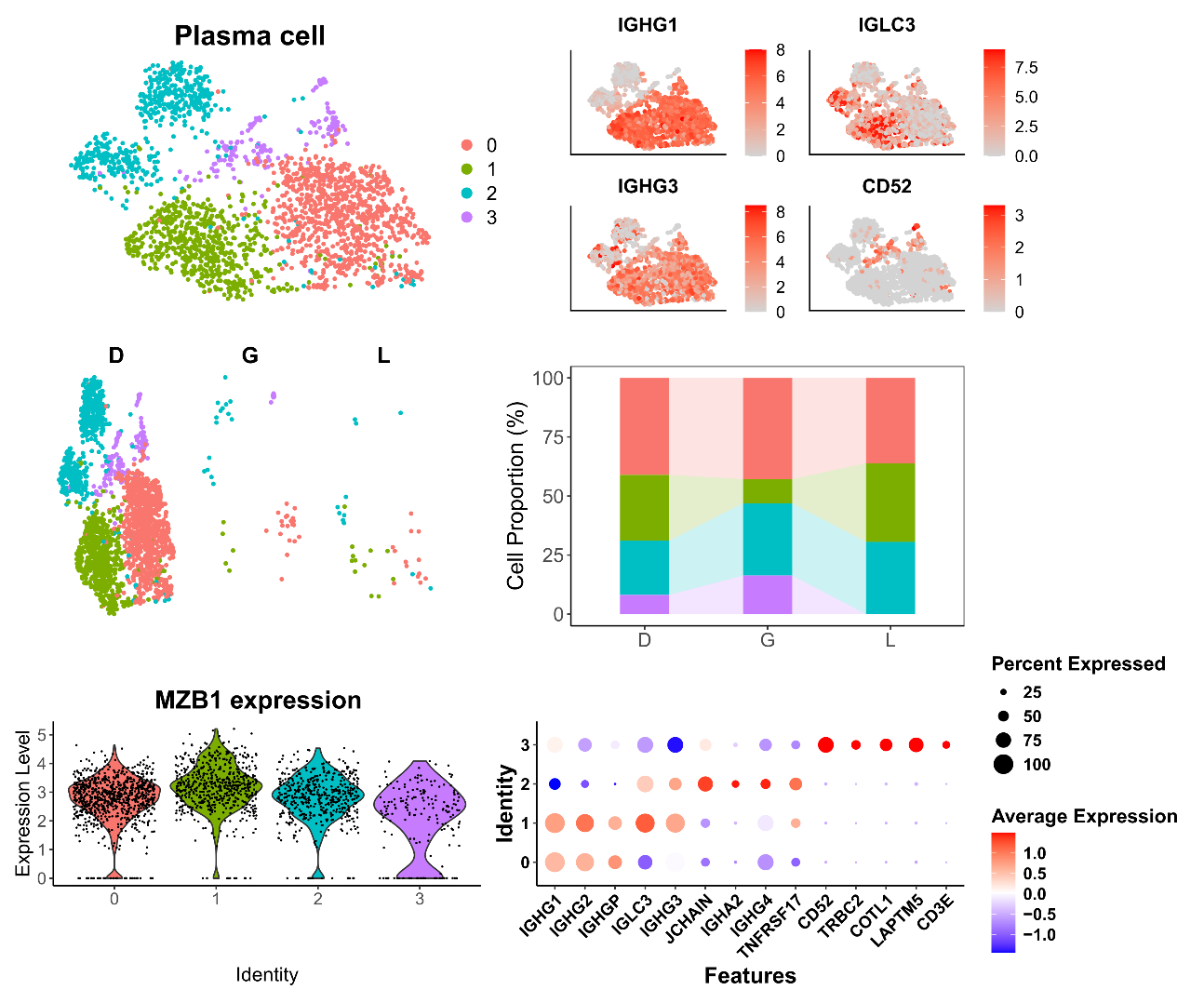


**Supplementary Figure 5. Plasma cell subcluster analysis**

UMAP projection of plasma cells showing tissue distribution with increased density in granulation samples. Selected markers correlation to UMAP to distinguish populations. Rate of cell annotation from each tissue resource. Dot plot showing high average expression of immunoglobulin constant regions and co-expression of regulatory genes (*TNFRSF17*, *JC HAIN*), consistent with active antibody production.


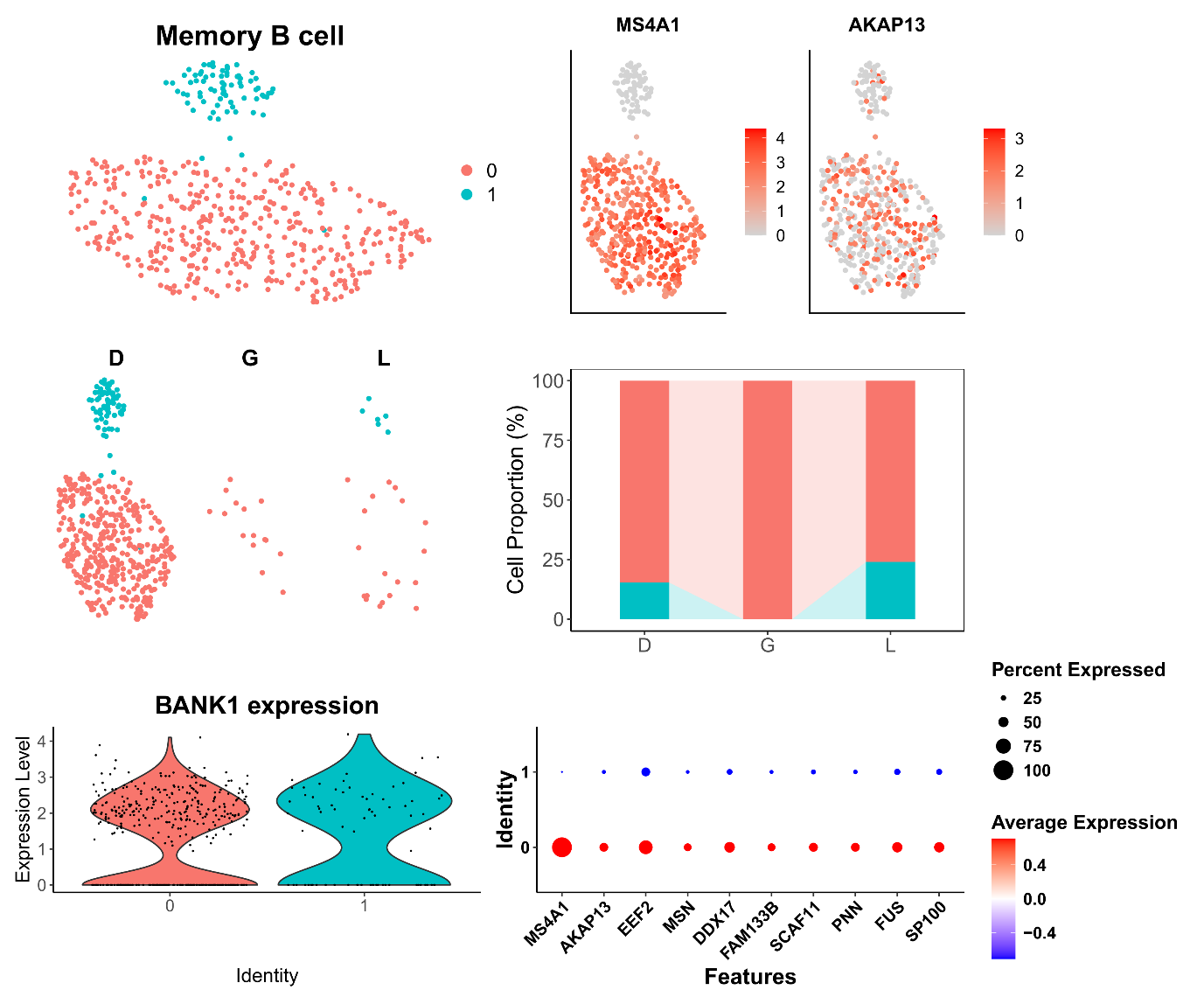


**Supplementary Figure 6. Memory B cell subcluster analysis**

UMAP projection of Memory B cell showing tissue distribution with increased density in granulation samples. Selected markers correlation to UMAP to distinguish populations. Rate of cell annotation from each tissue resource. Dot plot shows moderate expression of transcriptional regulators (*DDX17*, *FUS*, *SCAF11*).


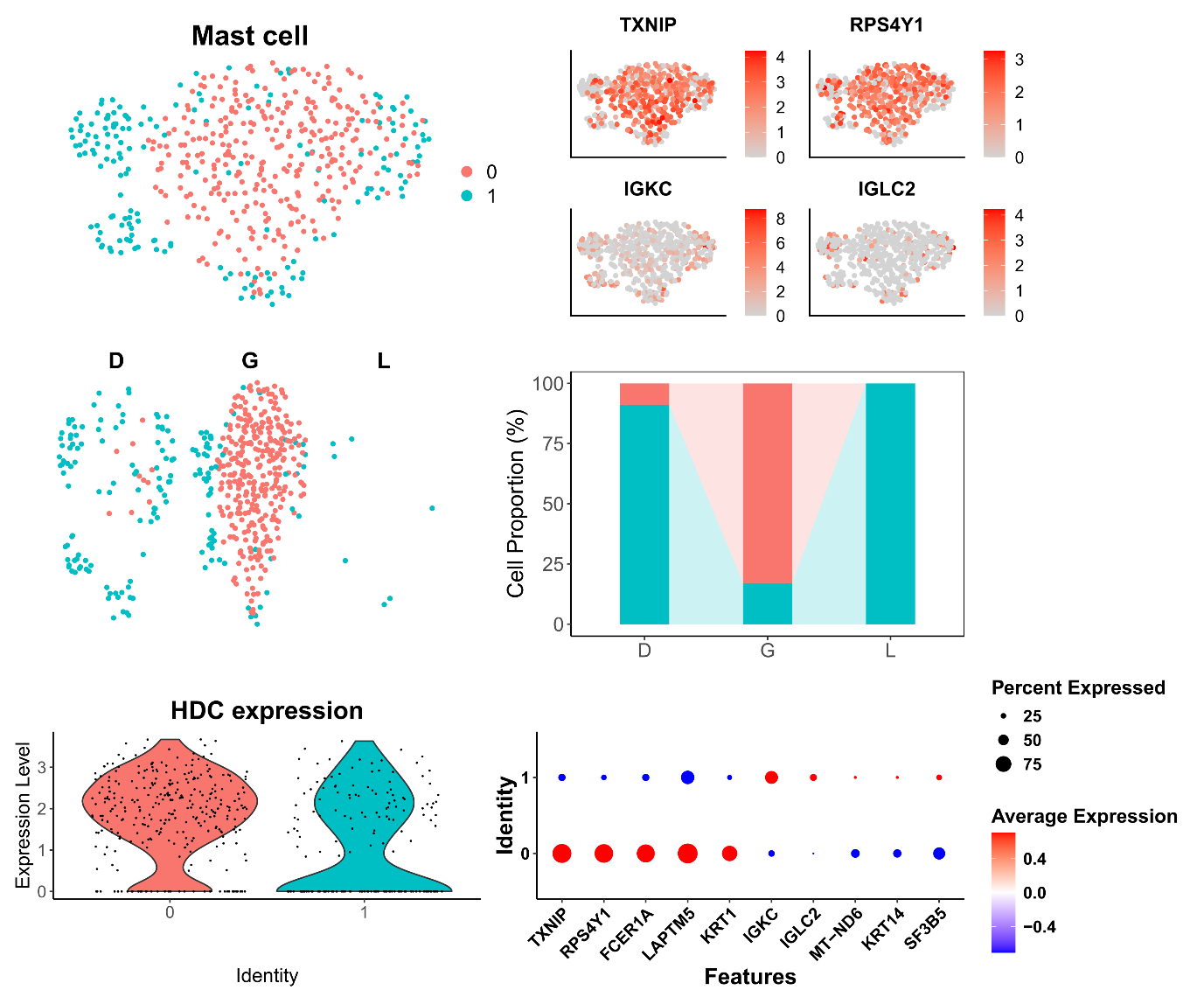


**Supplementary Figure 7. Mast cell subcluster analysis**

UMAP projection of Mast cell showing tissue distribution with increased density of population 1 of Mast cells in granulation samples. Selected markers correlation to UMAP to distinguish populations. Rate of cell annotation from each tissue resource. Dot plot shows high expression of *FCER1A*, *TXNIP* and *RPS4Y1* in population 0, whereas population 1 shows moderate expression of immunoglobulin transcripts (*IGKC*, *IGLC2*).


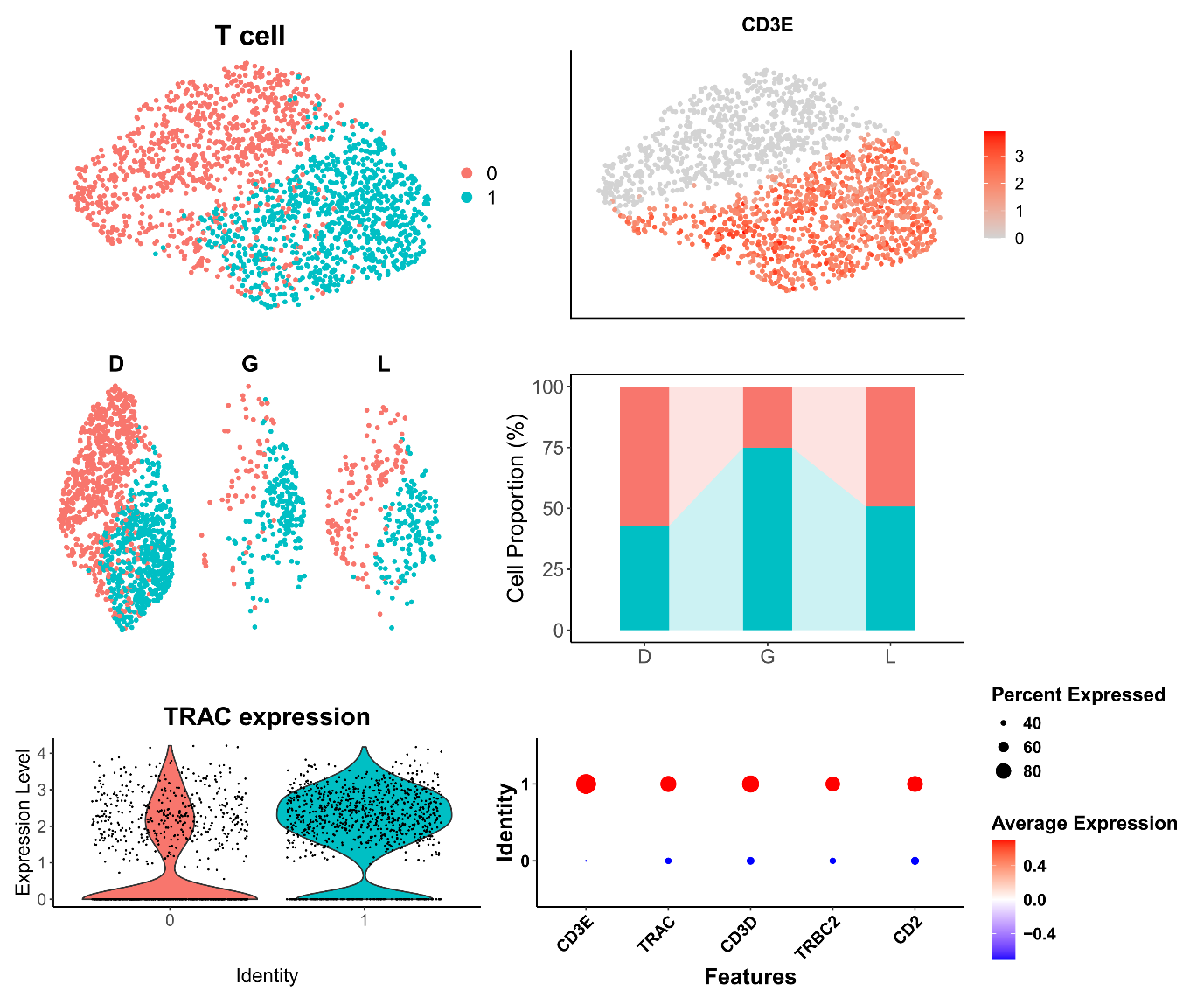


**Supplementary Figure 8. T cell subcluster analysis**

UMAP projection of T cell showing tissue distribution with increased density of population in granulation samples. Selected markers correlation to UMAP to distinguish populations. Rate of cell annotation from each tissue resource. Dot plot confirms expression of additional T cell markers (CD3E, *TRBC2*, *CD2, TRAC*) without strong evidence of subset-specific differentiation.


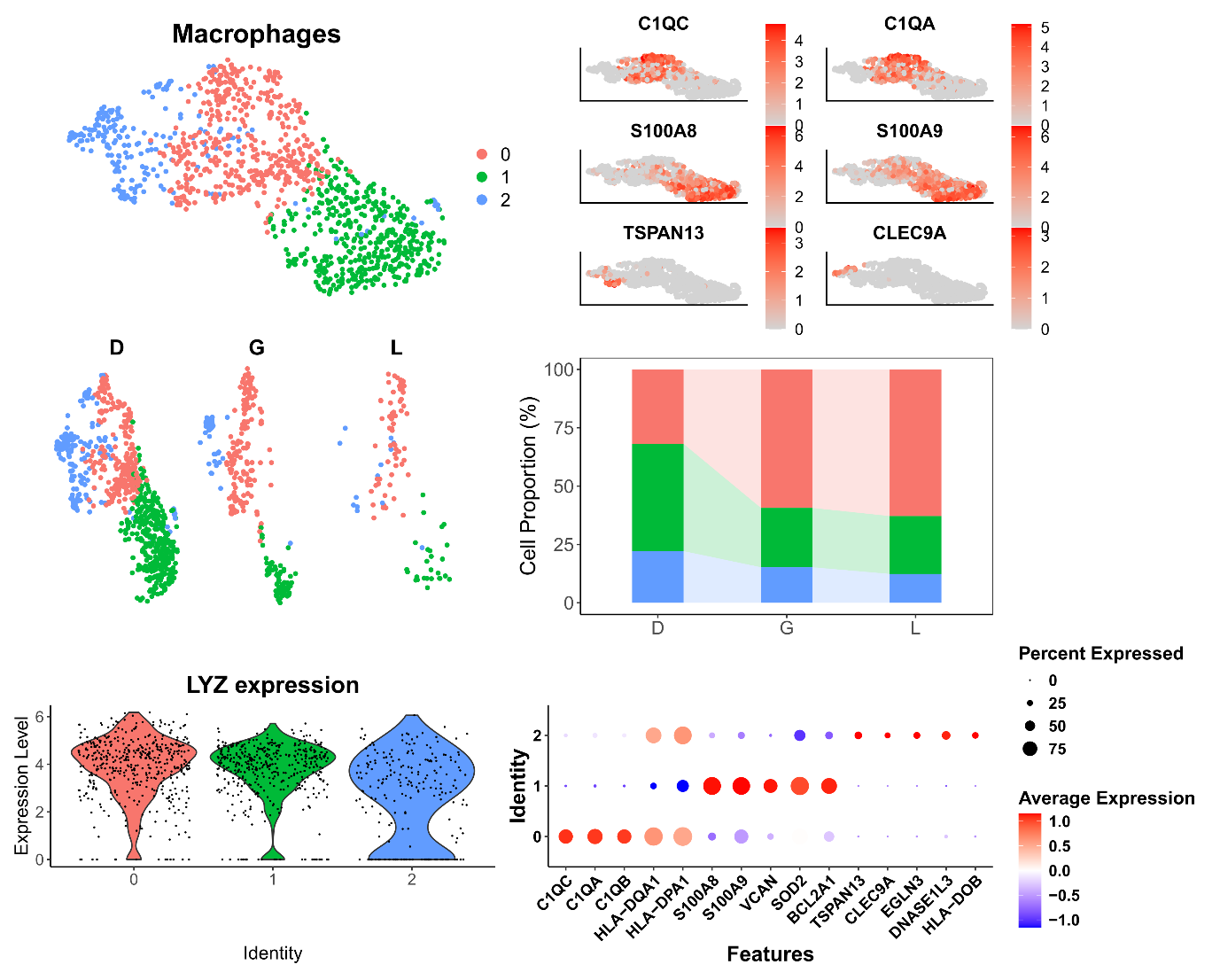


**Supplementary Figure 9. Macrophages subcluster analysis**

UMAP projection of Macrophages showing tissue distribution with increased density of population in granulation samples. Selected markers correlation to UMAP to distinguish populations. Rate of cell annotation from each tissue resource. Dot plot of differentially expressed genes of key macrophage markers, including *C1QA*, *C1QB*, *C1QC*, *S100A8*, *S100A9*, *VCAN*, *SOD2*, *HLA-DQA1*, *HLA-DPA1*, and *BCL2A1* indicating classical macrophage identity with partial activation signals.

**
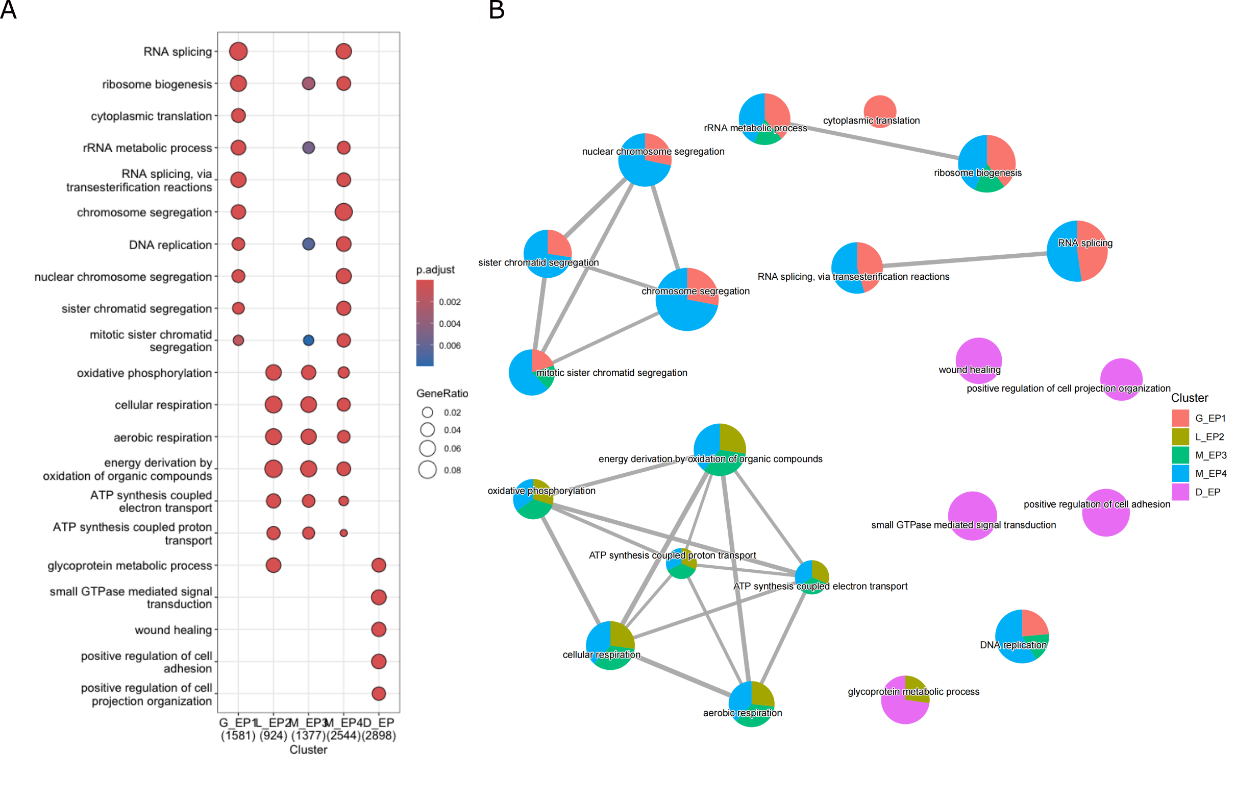
**

**Supplementary Figure 10. GO Analysis of Epithelial Cells**

(A) Gene Ontology (GO) enrichment analysis comparing functional differences across epithelial cell clusters, performed using the compareCluster function. (B) Visualization of GO enrichment results using the emapplot function from clusterProfiler, illustrating how each cluster contributes to enriched pathways.

**
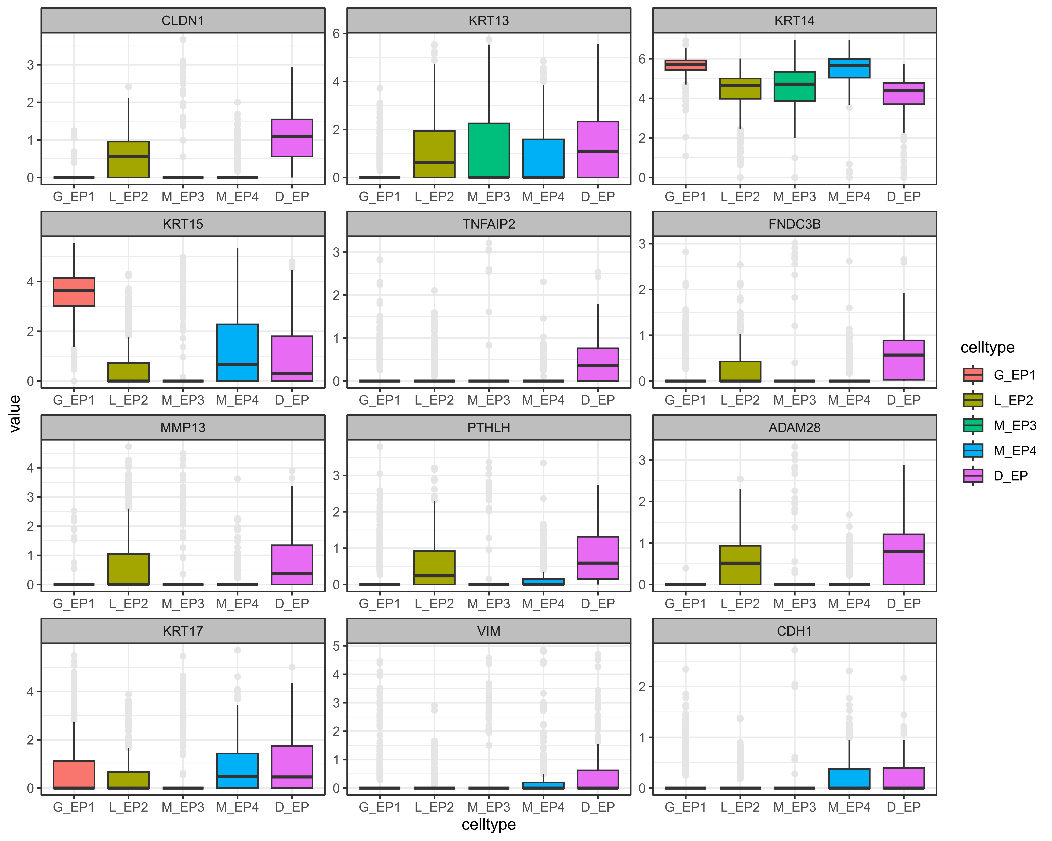
**

**Supplementary Figure 11. Relative Expression of Epithelial Cell Markers**

Bar plots showing the relative expression levels of typical epithelial cell markers across each cluster.

**
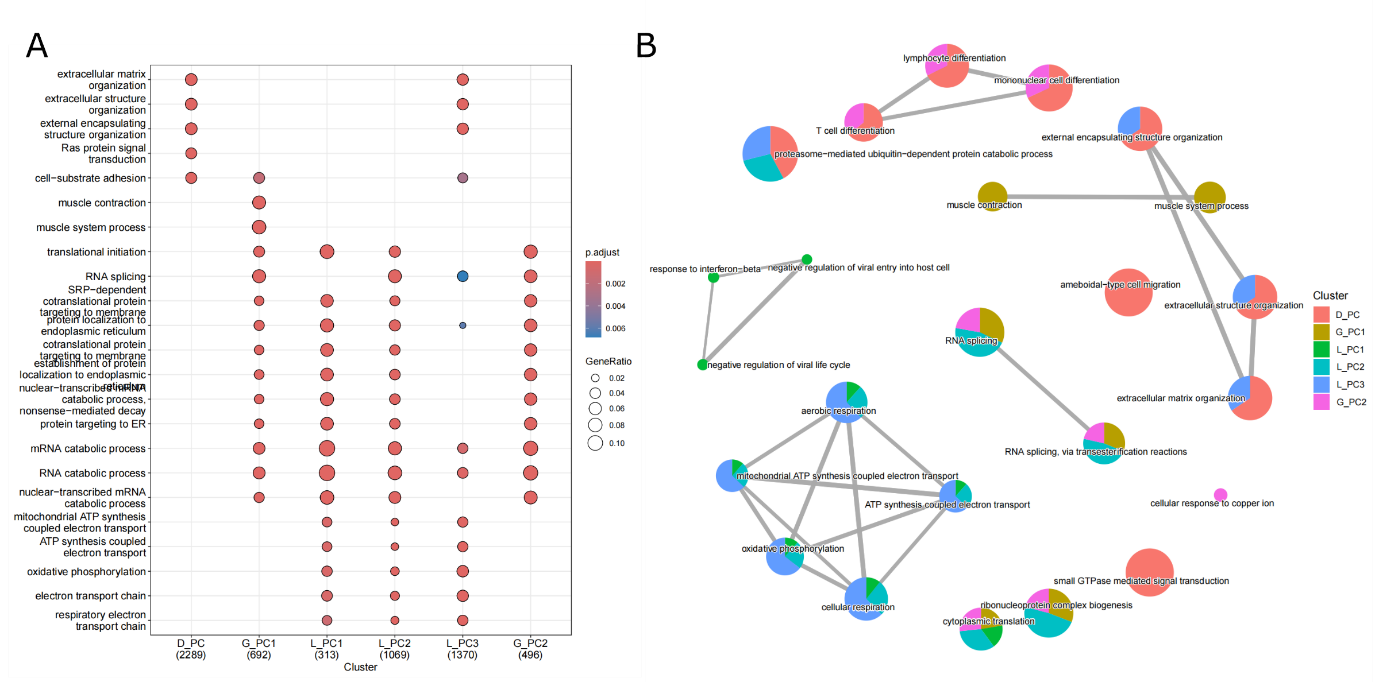
**

**Supplementary Figure 12. GO Analysis of Pericytes**

(A) Gene Ontology (GO) enrichment analysis comparing functional differences across Pericyte clusters, performed using the compareCluster function. (B) Visualization of GO enrichment results using the emapplot function from clusterProfiler, demonstrating contributions of each cluster to the enriched pathways.


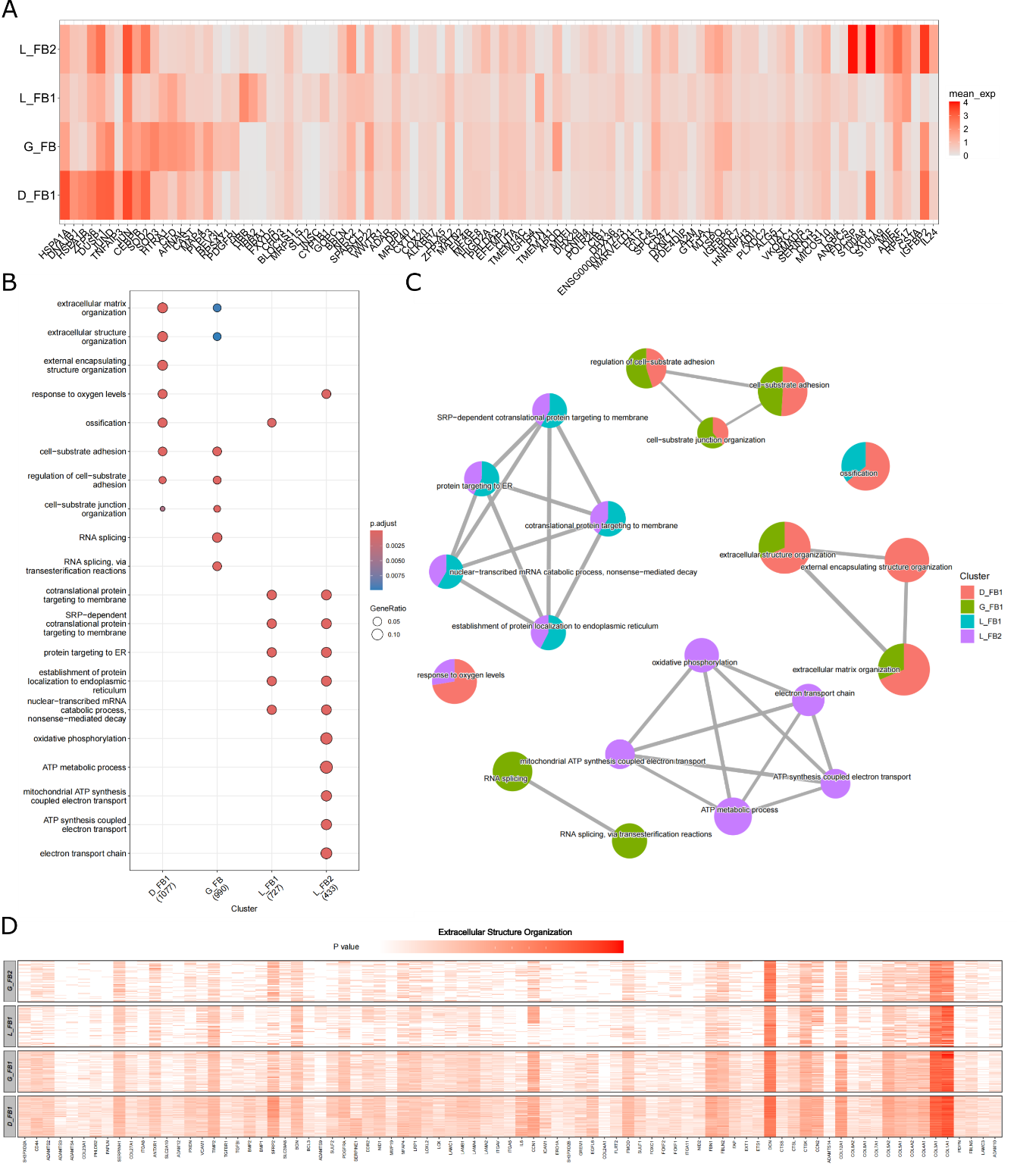


**Supplementary Figure 13**. **Gene expression and** **GO Analysis of Fibroblasts**

(A) Differentially expressed genes (DEGs) were normalized and visualized on a heatmap, displaying the Top 10 genes ranked by log2 fold change (log2FC). (B,C) GO enrichment terms for comparing clusters, using the compareCluster function, and emapplot function from clusterProfiler. (D) Extracelular Structure Organisation genes and their expression levels were plotted on a heatmap.


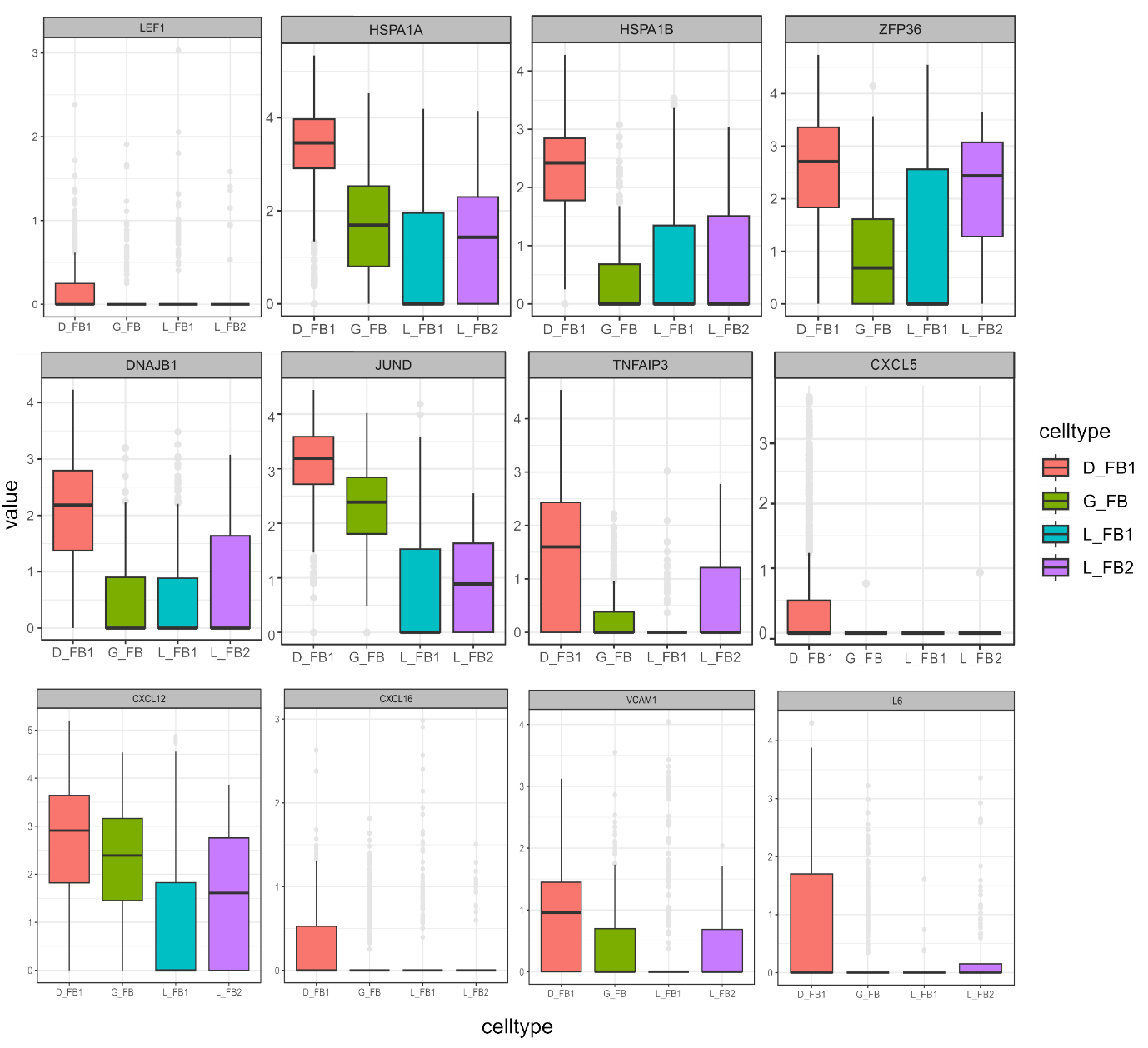


**Supplementary Figure 14. Bar plots showing the relative expression levels fibroblast cell markers across each cluster.**


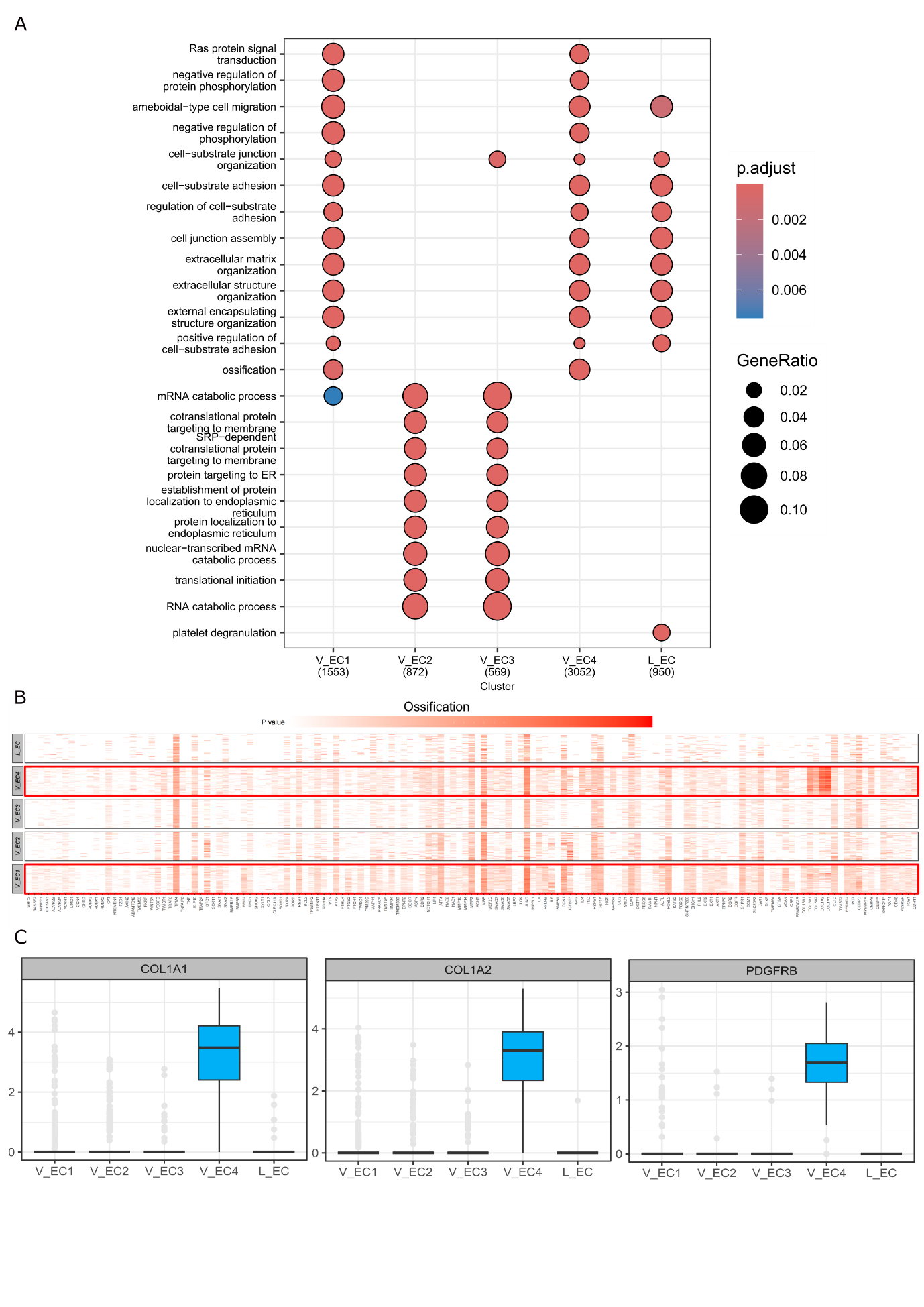


**Supplementary Figure 15. GO Analysis and Box Plot of Endothelial Cells**

(A) GO enrichment terms for comparing clusters, using the compareCluster function. (B) Ossification-enriched genes and their expression levels were plotted on a heatmap, where cluster VE_4 exhibited the highest expression levels of these genes. (C) Bar plots showing the relative expression of vascular development-related pathways in the endothelial subpopulations.


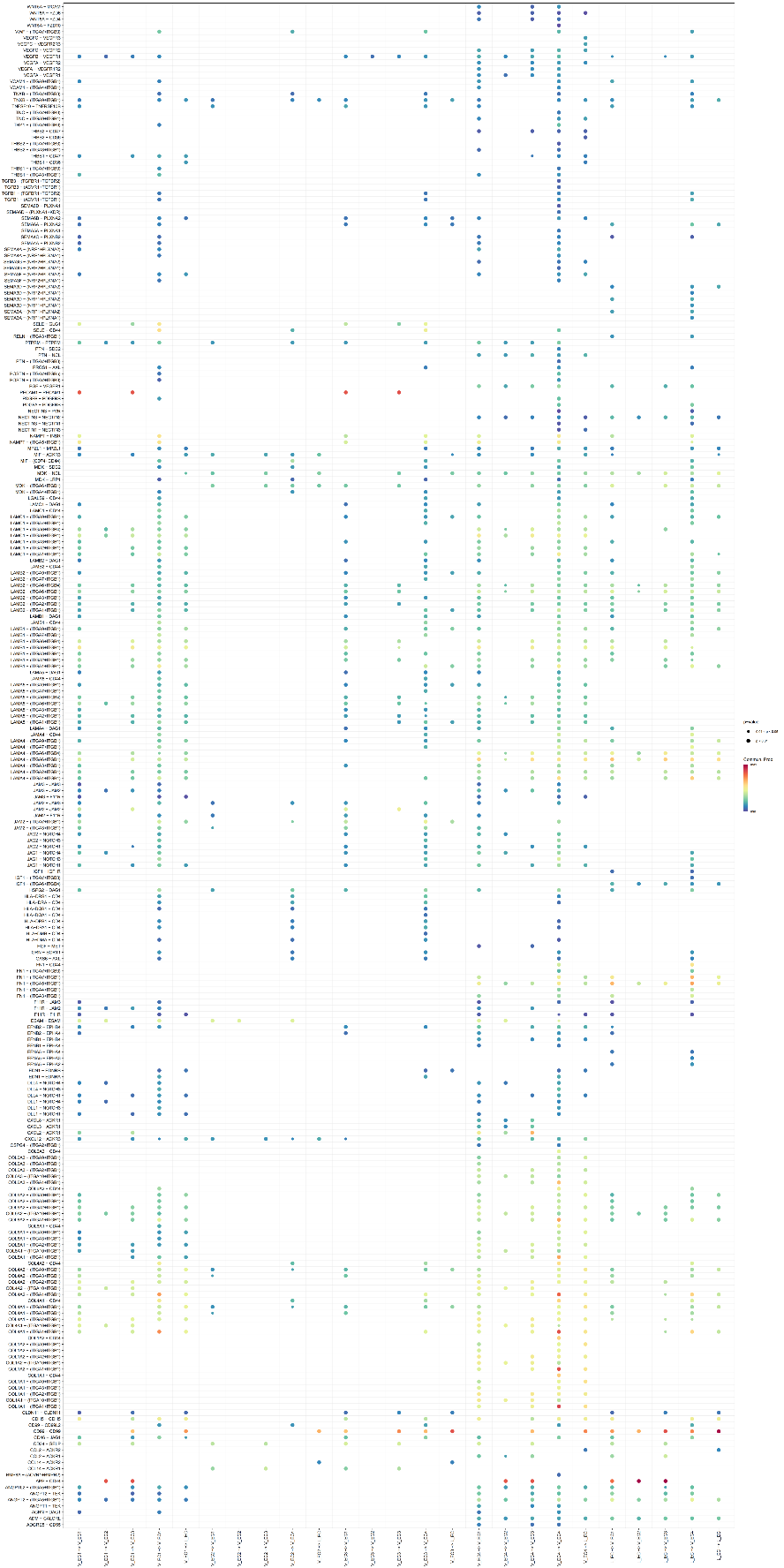


**Supplementary Figure 16.** The dot plot illustrates all significant ligand-receptor pairs contributing to signalling among endothelial subclusters in the dataset. The colour of each dot represents the calculated communication probability, while the size of the dot corresponds to the associated p-value. Empty spaces indicate instances where the communication probability is zero. P-values were computed using a one-sided permutation test.

**Supplementary materials and methods**

Tissue processing

Fresh tissue obtained during surgery from intrabony and suprabony defects were dissected and dissociated into a single cell suspension. Following mechanical dissociation with scalpel, the samples were placed in PBS + 2% FBS with 2 mg/ml of Collagenase Type II and 1 mg/ml of DNAse Type I (Merck), and incubated for 20–30 min at 37 °C shaking (120 rpm) with homogenization of the suspension every 3-4 minutes. After incubation, the cell suspensions were toped up to 12ml using using PBS + 2% FBS and filtered in using a 40µm cell strainer. These were subsequently centrifuged in 4 °C precooled centrifuge for 5 min at 300 × *g*. The samples were then cleansed for debris using Debris Removal Solution (Miltenyi Biotec) according to the manufacturer. The pellet was then re-suspended in 100 uL of PBS + Ultrapure^TM^BSA (0.04%) (Thermo-Fischer Scientific) and sent to the BRC Genomic Centre where the library preparation and sequencing were performed.

Single-cell suspensions were manually counted using a haemocytometer and concentration adjusted to a minimum of 300cells/μL. Cells were loaded according to standard protocol of the Chromium single-cell 3’ kit to capture around 5,000 cells per chip position. Briefly, a single-cell suspension in PBS 0.04% BSA was mixed with RT-PCR master mix and loaded together with Single Cell 3’ Gel Beads and Partitioning Oil into a Single Cell 3’ Chip (10x Genomics) according to the manufacturer’s instructions. RNA transcripts from single cells were uniquely barcoded and reverse transcribed. Samples were run on individual lanes of the Illumina HiSeq 2500.

Data Pre-processing and Quality Control

Raw sequencing data from granulation tissue, along with datasets from GSE152042 (gingiva)[1] and GSE161267 (periodontal ligament) [2], were demultiplexed and mapped to the human reference genome (GRCh38) using Cell Ranger 6 (v5.0) [3]. Gene expression matrices were generated and processed for quality control in RStudio (v4.1.2) using Seurat (v5.1.0) [4]. During quality control, genes expressed in fewer than three cells were removed. Cells with more than 8,000 unique feature counts or fewer than 600 unique feature counts were excluded to filter out potential doublets, poor-quality cells, or empty droplets. Additionally, cells with mitochondrial gene expression exceeding 25% were removed to ensure data integrity. After pre-processing, the dataset consisted of 6,729 cells from disease granulation tissue ('D'), 4,001 cells from gingiva ('G'), and 3,024 cells from periodontal ligament ('L').

Bioinformatics Analysis of Integrated scRNAseq Datasets

Based on the filtered cells, data normalization was first performed to adjust for sequencing depth. The 2,000 most variable features in each dataset were identified using the variance-stabilizing transformation method. Next, integration anchors were computed across the datasets, and batch effects were removed through data integration. This integration approach was carefully compared with a non-integration strategy to assess alignment with known subcluster characteristics. Differences between clusters were attributed to tissue-specific gene expression variations rather than batch effects, based on the subcluster analysis, thereby ensuring the biological accuracy of the findings.

Following these steps, the data were scaled to account for sequencing depth and other technical variations. Principal component analysis (PCA) was performed to identify key dimensions for clustering. A k-nearest neighbor (KNN) graph was constructed to establish relationships between cells, and clustering was carried out using the Louvain algorithm, optimizing modularity with a resolution parameter of 0.5. To visualize cell distribution, uniform manifold approximation and projection (UMAP) was applied, focusing on the first 30 principal components and incorporating 30 nearest neighbors. Marker genes were then identified by analysing differential expression using the non-parametric Wilcoxon rank sum test, as implemented in Seurat's default FindMarkers function.

Cell type annotation was performed based on the expression of specific marker genes within each cluster. Cell types identified from our datasets included plasma cells (MZB1), endothelial cells (EMCN, CLDN5), T cells (TRAC, TRBC1), epithelial cells (KRT15, KRT14), fibroblasts (COL1A1, COL6A1, FAP), pericytes (RGS5, ACTA2, FRZB), macrophages (LYZ), memory B cells (BANK1), and mast cells (HDC). The labelled UMAP was visualized either on the merged dataset or on each of the three tissues individually. Additionally, the percentage of each cell type was calculated and clustered across the tissues.

Subcluster re-clustering

Macrophages, Mast cells, Memory B cells, Plasma cells, T cells, Endothelial, Epithelial, Fibroblast, and Pericytes populations were identified within their respective clusters in the UMAP plot and were subsequently re-clustered following the same protocol outlined above for the second round of analysis. For this re-clustering, UMAP was utilized with a resolution parameter set to 0.25.

Gene Ontology (GO) analysis

The ClusterProfiler package in R [5] was used for functional enrichment analysis using the molecular functions and biological pathways gene ontology (GO) annotations and Kyoto Encyclopedia of Genes and Genomes (KEGG) pathways. The enrichment addressed the marker list of each cluster (adjusted for p < 0.05).

Cell-Cell Communication

Cell-cell communication within the subclusters was analysed using the CellChat pipeline (v1.1.3) [6]. A new CellChat object was created from the merged Seurat object, and the complete CellChat database, including ECM signalling, cell-cell contact, and secreted signalling pathways, was selected for analysis. Communication probabilities were computed using a truncated mean approach (computeCommunProb function, type = "truncatedMean", trim = 0.2). Subsequently, a cell-cell communication network was inferred, and downstream visualization techniques were applied to depict the interactions.

Single-cell trajectory analysis

Pseudotime analysis was performed using Monocle2 [7] with the DDR-Tree algorithm and default parameters, marker genes for each subcluster were selected, and raw expression counts from cells that passed quality control filtering were utilized, then, the analysis was conducted to model the differentiation trajectories, and Branch Expression Analysis Modeling (BEAM) was applied to identify genes associated with branch-specific fate decisions. The starting point of the pseudotime trajectory was determined using CytoTRACE2 [8], based on the differentiation potential predicted by the model. This integrated approach enabled the assessment of differentiation levels among clusters and validated the pseudotime starting point by integrating marker genes that exhibited differential expression according to our list of marker genes.

Gene Set Enrichment Analysis (GSEA)

Differentially expressed genes that induced functional pathways between endothelial cells from different sources were compared, with contrasts made between "D" versus "L" and "D" versus "G". Gene Set Enrichment Analysis (GSEA, v4.0.3) [9] was performed to evaluate endothelial cell-induced vascular development between granulation and healthy tissues. The top 5 enriched pathways were visualized using a dot plot, with rankings based on the enrichment P-value.

Histology

Granulation samples collected were fixed in 4% paraformaldehyde (PFA) for 24 hours at 4°C. The samples were processed and mounted in O.C.T. and cut in the cryostat (BRIGHT, OTF5000) at 8μm thickness. To visualise the tissue morphology, the slides were stained using Haematoxylin & Eosin staining and viewed in a light-field Nikon Eclipse Ci-L microscope.

Immunofluorescence

To visualise and validate the findings of the sequencing, the samples were stained with mouse polyclonal anti-VWF antibody (1:100; Abcam; ab201336), rabbit polyclonal anti-RGS5 antibody (1:50; Thermofisher; 11590-1-AP), rabbit polyclonal anti-NOTCH3 antibody (1:250; Abcam; ab23426), rabbit polyclonal anti-HEY1 antibody (1:50; Abcam; ab154077) overnight at 4 °C. Sections were washed and exposed to secondary antibody (1:250; Thermo Fisher Scientific; A21449) for 1 h at room temperature. Imaging was generated using a Thunder microscope.

1. Caetano, Ana J., Val Yianni, Ana Volponi, Veronica Booth, Eleanor M. D’Agostino, and Paul Sharpe. 2021. ‘Defining Human Mesenchymal and Epithelial Heterogeneity in Response to Oral Inflammatory Disease’. *eLife* 10:e62810. doi: [10.7554/eLife.62810](https://doi.org/10.7554/eLife.62810).
2. Pagella, Pierfrancesco, Laura De Vargas Roditi, Bernd Stadlinger, Andreas E. Moor, and Thimios A. Mitsiadis. 2021. ‘A Single-Cell Atlas of Human Teeth’. *iScience* 24(5):102405. doi: [10.1016/j.isci.2021.102405](https://doi.org/10.1016/j.isci.2021.102405).
3. Zheng, Grace X. Y., Jessica M. Terry, Phillip Belgrader, Paul Ryvkin, Zachary W. Bent, Ryan Wilson, et al. 2017. ‘Massively Parallel Digital Transcriptional Profiling of Single Cells’. *Nature Communications* 8(1):14049. doi: [10.1038/ncomms14049](https://doi.org/10.1038/ncomms14049).
4. Hao, Yuhan, Tim Stuart, Madeline H. Kowalski, Saket Choudhary, Paul Hoffman, Austin Hartman, Avi Srivastava, Gesmira Molla, Shaista Madad, Carlos Fernandez-Granda, and Rahul Satija. 2024. ‘Dictionary Learning for Integrative, Multimodal and Scalable Single-Cell Analysis’. *Nature Biotechnology* 42(2):293–304. doi: [10.1038/s41587-023-01767-y](https://doi.org/10.1038/s41587-023-01767-y).
5. Xu, Shuangbin, Erqiang Hu, Yantong Cai, Zijing Xie, Xiao Luo, Li Zhan, Wenli Tang, Qianwen Wang, Bingdong Liu, Rui Wang, Wenqin Xie, Tianzhi Wu, Liwei Xie, and Guangchuang Yu. 2024. ‘Using clusterProfiler to Characterize Multiomics Data’. *Nature Protocols* 19(11):3292–3320. doi: [10.1038/s41596-024-01020-z](https://doi.org/10.1038/s41596-024-01020-z).
6. Jin, Suoqin, Christian F. Guerrero-Juarez, Lihua Zhang, Ivan Chang, Raul Ramos, Chen-Hsiang Kuan, Peggy Myung, Maksim V. Plikus, and Qing Nie. 2021. ‘Inference and Analysis of Cell-Cell Communication Using CellChat’. *Nature Communications* 12(1):1088. doi: [10.1038/s41467-021-21246-9](https://doi.org/10.1038/s41467-021-21246-9).
7. Qiu, Xiaojie, Qi Mao, Ying Tang, Li Wang, Raghav Chawla, Hannah A. Pliner, and Cole Trapnell. 2017. ‘Reversed Graph Embedding Resolves Complex Single-Cell Trajectories’. *Nature Methods* 14(10):979–82. doi: [10.1038/nmeth.4402](https://doi.org/10.1038/nmeth.4402).
8. Kang, Minji, Jose Juan Almagro Armenteros, Gunsagar S. Gulati, Rachel Gleyzer, Susanna Avagyan, Erin L. Brown, Wubing Zhang, Abul Usmani, Noah Earland, Zhenqin Wu, James Zou, Ryan C. Fields, David Y. Chen, Aadel A. Chaudhuri, and Aaron M. Newman. 2024. ‘Mapping Single-Cell Developmental Potential in Health and Disease with Interpretable Deep Learning’. bioRxiv [Preprint]. Mar 21:2024.03.19.585637. doi: 10.1101/2024.03.19.585637.
9. Subramanian, Aravind, Pablo Tamayo, Vamsi K. Mootha, Sayan Mukherjee, Benjamin L. Ebert, Michael A. Gillette, Amanda Paulovich, Scott L. Pomeroy, Todd R. Golub, Eric S. Lander, and Jill P. Mesirov. 2005. ‘Gene Set Enrichment Analysis: A Knowledge-Based Approach for Interpreting Genome-Wide Expression Profiles’. *Proceedings of the National Academy of Sciences* 102(43):15545–50. doi: [10.1073/pnas.0506580102](https://doi.org/10.1073/pnas.0506580102).
